# Research progress on multimodal data fusion in forest resource monitoring

**DOI:** 10.3389/fpls.2025.1710618

**Published:** 2026-01-19

**Authors:** Ming Wang, Qian Zhang, Xin Liu, Jinmeng Zhang, Feng Yu, Xining Zhang, Ruifang Zhao

**Affiliations:** Institute of Data Science and Agricultural Economics, Beijing Academy of Agriculture and Forestry Sciences, Beijing, China

**Keywords:** deep learning, forest resource monitoring, fusion strategy, multimodal data fusion, preprocessing

## Abstract

Dynamic monitoring of forest resources is crucial for safeguarding global ecological security. However, traditional monitoring methods, limited by single data sources, struggle to meet the demands of refined management. The global forest loss area in 2024 surged by 80% compared with that in 2023, further highlighting the urgency of technological upgrading. Multimodal data fusion technology has emerged as a core solution by establishing an “air-space-ground” collaborative network integrating “satellite remote sensing (macro-scale) + UAV hyperspectral (meso-scale) + ground sensors (micro-scale)”. This technology integrates multi-source heterogeneous data such as optical, radar, and LiDAR data, and achieves cross-modal information complementarity by combining traditional machine learning and deep learning. Based on the framework of “technical characteristics-scenario applications-challenge breakthroughs”, this study systematically reviews the research progress from 2020 to 2025. Technically, a complete technology chain is established, covering data acquisition, data preprocessing (including key links such as “data cleaning-spatiotemporal registration-feature dimensionality reduction”), and multi-strategy fusion. Significant application effects have been achieved in scenarios including tree species classification, land resource monitoring, forest structure parameter estimation and ecological monitoring, as well as forest disaster monitoring and tree health assessment. Meanwhile, the study identifies key technical bottlenecks: in data acquisition, the accuracy of LiDAR point clouds in dense forest areas decreases by 15%-20%; in preprocessing, issues such as spatiotemporal registration errors and high annotation costs exist; in fusion strategies, the accuracy of early fusion decreases by 12% when the number of features exceeds 500 dimensions; in model deployment, the inference latency of edge devices increases by 20%-30%. The core contributions of this study are as follows: constructing a standardized “air-space-ground” data technology chain, proposing a scenario-adaptable fusion framework, and clarifying future directions such as model lightweighting and edge computing. These contributions provide support for the engineering application of this technology and promote the transformation of forestry monitoring from “experience-driven” to “intelligent data-driven”.

## 1 Introduction

As a core component of the global ecosystem, forests are not only the “carbon sink hub” that maintains the balance of the carbon cycle but also the “natural gene bank” that supports biodiversity. Their dynamic changes are directly related to global climate change and ecological security (Alfieri et al., 2024; Balestra et al., 2025). In recent years, frequent ecological disasters have still exposed the vulnerability of forest ecosystems. According to the recently released data by the “Global Forest Watch Think Tank”, the global forest loss area in 2024 was 80% more than that in 2023 (WRI, 2025). This severe reality has exposed the deep-seated shortcomings of traditional monitoring systems, which are difficult to support the needs of refined management.

Traditional monitoring methods have significant technical limitations: optical remote sensing (such as Sentinel-2) is susceptible to interference from cloud cover and vegetation occlusion, resulting in a low capture rate of the real state of complex forest areas; although LiDAR can obtain three-dimensional structural information, its cost is relatively high, and the coverage rate of large-scale applications is insufficient; manual patrol has high cost and low efficiency, and remote forest areas are basically in the monitoring blind spot. The “information island” of a single data source and the “full-dimensional monitoring demand” of complex ecological scenarios form a sharp contradiction, which is far from meeting the practical needs of carbon sink accounting and disaster early warning. Against this background, the multimodal data fusion technology has broken the limitations of data sources by constructing an air-space-ground collaborative network of “satellite remote sensing (macroscopic wide area) + UAV hyperspectrum (mesoscopic details) + ground sensors (microscopic dynamics)”. This technology is not a simple superposition of information, but explores the complementary correlations among spectral, structural, and meteorological data through deep learning algorithms (such as cross-attention networks). This value chain “from data collaboration to decision-making upgrading”(Y. Li et al., 2022; Su et al., 2023) offers a core solution for the refined management of forest resources. its practical application in forest monitoring is confronted with distinctive challenges. Forests’ complex structures, diverse species, and dynamic environments, such as frequent cloud cover affecting optical data acquisition and the difficulty in aligning spectral and LiDAR features for heterogeneous tree species, pose hurdles. These challenges will be examined in subsequent sections to better realize the technology’s value.

**Figure 1:**
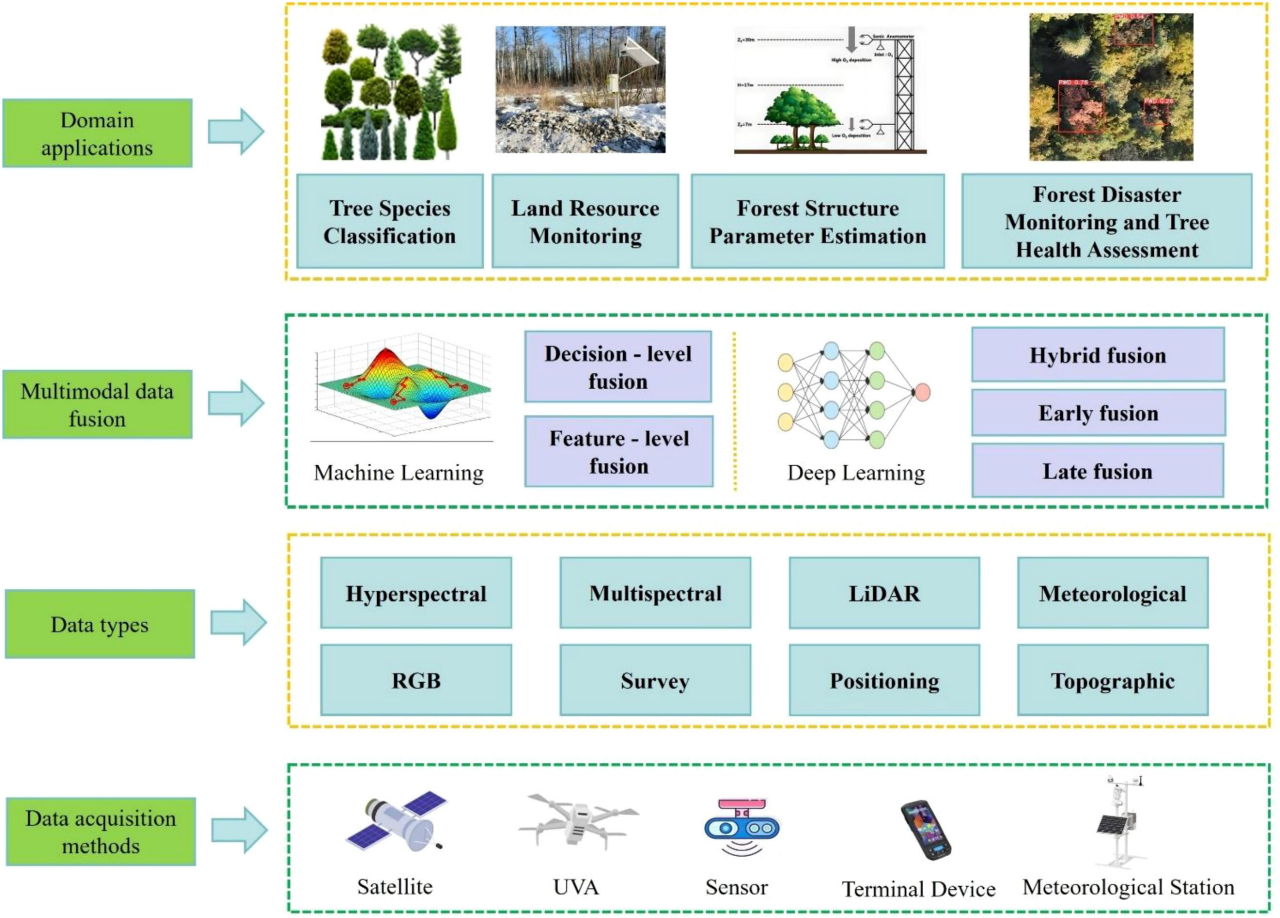
Overall structure of the thesis.

To systematically synthesize the research progress of multimodal technology in the field of forest resource monitoring over the past 5 years (2020–2025), this study has adopted a systematic review method to design the literature retrieval and screening strategy. The retrieval has taken the Web of Science Core Collection (including SCI-E and SSCI) as the main database, with the Scopus database supplemented simultaneously to avoid literature omission. For keywords, a combination of “subject terms + synonym expansion” has been used, covering keywords such as “multimodal data fusion”, “cross-modal fusion”, “multi-modality fusion”, “forest resource monitoring”, “forest ecosystem monitoring”, “forest inventory”, “remote sensing”, “LiDAR”, and “UAV hyperspectral”, so as to ensure the inclusion of studies related to relevant technologies and scenarios. The literature screening process has consisted of three steps: first, 2,276 literatures were initially retrieved, and 1,552 were retained after removing duplicates; second, irrelevant studies were excluded through title/abstract screening, leaving 489 literatures; finally, literatures inconsistent with the required technical methods were eliminated through full-text verification, resulting in the final literatures for analysis and citation. This study has also constructed a three-dimensional analytical framework of “technical characteristics - scenario application - challenge breakthrough”. Firstly, in Chapter 2, the multimodal technology system has been analyzed from three dimensions (data sources, preprocessing, and fusion strategies), laying a technical foundation for the research. Then, in Chapter 3, the aforementioned technologies have been combined with forestry scenarios such as tree species classification and land resource monitoring, and the technical adaptability and application effects have been verified quantitatively through cases to reflect scenario applications. In the last two chapters, solutions at the data, algorithm, and application levels have been proposed for technical shortcomings and scenario pain points, and future research directions have been clarified, which has helped to promote the transformation of forestry monitoring from “experience-driven” to “intelligent data-driven”. **Figure 1** illustrates the overall structure of the thesis, encompassing data acquisition methods, data types, multimodal data fusion approaches, and their corresponding domain applications in forestry.

## 2 Multimodal data technology characteristics

### 2.1 Typical applications of multimodal data fusion in agricultural production

Multimodal data fusion has been widely used in agricultural production, covering scenarios such as yield measurement (Ali et al., 2022), crop classification (Chakhar et al., 2021), production refinement monitoring (Peng et al., 2024; Xu et al., 2022), intelligent operation (Borz and Proto, 2024), farmland quality assessment (Duan et al., 2022), and pest and disease detection (Zhang et al., 2023a; Zhou et al., 2021). By integrating multiple types of data sources, including satellite remote sensing, UAV remote sensing, meteorological observation, and soil monitoring (with their data formats covering texts, images, etc.), and combining model algorithms adapted to different data characteristics—such as U-Net, Random Forest (RF), Deep Neural Network (DNN), and eXtreme Gradient Boosting (XGB)—the information complementarity of multi-source data and the synergistic improvement of model performance are effectively achieved.

The mainstream paradigm of “Remote Sensing Imagery + Ground Measurement + Environmental Data” is formed in yield measurement, which integrates RGB images (Lv et al., 2024) multispectral/hyperspectral imagery, LiDAR point clouds and meteorological/soil data, and with the help of algorithms such as PLSR, SVR, RFR, DNN, etc., to capture complex linear relationships between yield and multiple factors. The algorithms such as PLSR, SVR, RFR, DNN, etc. were used to capture the complex nonlinear relationship between yield and multiple factors. Extensive research has been carried out in cotton (Mitra et al., 2024), soybean (Maimaitijiang et al., 2020; Yi Zhang et al., 2023b), tea (Ramzan et al., 2023), corn (W. Zhou et al., 2023), wheat (T. Cheng et al., 2024; Fei et al., 2023; Ma et al., 2023), etc. These applications comprehensively capture the complex relationship between the environment, crop phenotypes (Y. Wang et al., 2024a), and yield in agricultural production, and exhibit significant advantages in complementary feature enhancement, spatiotemporal dynamic capture, and adaptive weight optimization (Yuan et al., 2024).

In the aspects of crop classification and refined monitoring of production, the synergy of multi-source data can significantly improve accuracy (Shuai et al., 2024). proposed two innovative decision fusion strategies, Enhanced Overall Accuracy Index (E-OAI) and Majority Voting based on Overall Accuracy Index (OAI-MV), integrating multi-source remote sensing data with multiple classifiers, which significantly improved the accuracy of crop and vegetation classification; (T. Cheng et al., 2024) proposed a yield assessment method for wheat based on multimodal and time series networks, combined with LSTM model for yield prediction of different heat-tolerant genotypes of wheat. modal and time series network-based wheat yield assessment method, combined with the LSTM model for yield prediction of different heat-tolerant genotypes of wheat, resulted in an overall improvement of yield prediction accuracy (R²) of about 0.07. (S. Zhang et al., 2025) utilized UAV multi-source (texture characteristics, vegetation index, heat index) and multi-stage (bud, flowering, boll stage, fluffing) data to estimate cotton water content (CWC) to address the monitoring limitations of the traditional methods.

Multimodal data fusion also plays a role in agricultural land quality assessment, pest and disease detection and other needs. (L. Li et al., 2023) integrates satellite remote sensing, environmental and socio-economic multimodal data through the Google Earth Engine platform, and constructs random forest (RF) and deep neural network (DNN) models, with the best prediction performance of multimodal data combination; (J. Duan et al., 2023) constructs a multimodal framework that integrates text semantics (Tiny-BERT) and image features (R-CNN+ResNet-18), and the weighted average model AUC reaches 0.994, which improves the accuracy of agricultural pest detection; (Gopi and Karthikeyan, 2023) develops a multimodal machine learning crop recommendation and yield prediction model (MMML-CRYP), and the accuracy rate of crop recommendation exceeds 97%, which comprehensively captures the complex relationship between environment, crop phenotype and yield in agricultural production. **Table 1** presents the typical applications of multimodal data fusion in different agricultural production scenarios (such as yield measurement, crop classification, etc.), including the data sources used, model algorithms, and achieved effects.

**Table 1:** Typical applications of multimodal data fusion in agricultural production.

Literature number	Data source	Model algorithm	Application area	Optimal effect	Year
(S. Zhang et al., 2025)	Textural, VIs, TIs	PLSR, SVR, RFR, XGB	Cotton moisture content (CWC) estimation	R² is 0.860	2025
(Maimaitijiang et al., 2020)	RGB, Multispectral, Thermal infrared	DNN	Soybean Production Measurement	R² is 0.720 and RMSE is 15.99%	2020
(Ramzan et al., 2023)	Meteorological, Landsat-8	DNN	Tea production measurement	R² is 0.99	2023
(T. Cheng et al., 2024)	Multispectral, RGB	LSTM	Wheat production forecasts	R² is improved by approximately 0.07 overall.	2024
(Shuai et al., 2024)	GF-6, Sentinel-1, Sentinel-2	U-Net, OAI-MV	Crop/vegetation classification	The highest overall accuracy reaches 91.49%.	2024
(L. Li et al., 2023)	Satellite remote sensing, Environmental data, Socio-economic data	RF, DNN	Agricultural land quality assessment	Multimodal data fusion captures over 85% of quality variation	2023
(J. Duan et al., 2023)	Text, Image data	Tiny-BERT, R-CNN+ResNet-18	Pest and disease detection and classification	The weighted average model AUC reached 0.99	2023
(Gopi and Karthikeyan, 2023)	Soil nutrients, Environmental data, Production data	MMML-CRYP	Crop Recommendations	Crop recommendations are 97.91% accurate	2023

### 2.2 Forestry multi-source data sources and methods

Forestry data sources are rich and diversified (Zou et al., 2019), covering Space-borne remote sensing (satellite remote sensing to acquire raster data and extract spectral information), low-altitude near-earth (drones carrying payloads to acquire raster images and extract landscape texture), Field inventory (manual field measurement of structured/semi-structured data to acquire information on forests, etc.), localization and topography (using GPS and other means to acquire vector data to clarify spatial location, etc.), dynamic monitoring (WSN collects data to capture the dynamics of the forest microenvironment), meteorological data (data supplied by meteorological departments/stations to reflect regional climate factors). Among them, UAV data acquisition has the advantage of high spatial and temporal resolution, flexible operation, suitable for small and medium-sized dynamic monitoring (Istiak et al., 2023), combined with multi-sensors can collect multi-dimensional information (Jurado et al., 2022); Remote sensing data (spac-based and low-altitude), is a key support for grasping the distribution, growth status and dynamic changes of forest resources, and helps precise forestry management and decision-making, relying on wide-area coverage and technologies such as multi-spectral/radar. **Table 2** classifies and summarizes forestry multimodal data, clarifying the acquisition methods, data forms, data details, and extractable information for each type of data.

**Table 2:** Forestry multimodal data classification and technical characteristics.

Data classification	Acquisition method	Data format	Breakdown of data	Extracted information
Space-borne remote sensing	Satellite remote sensing (John and Zhang, 2022)	Raster data	Optical remote sensing (multispectral/hyperspectral), radar remote sensing, LiDAR data	Spectral information, vegetation index, forest vertical structure, 3D point cloud, Phenotypic information, three-dimensional structure of canopy
Low altitude near-Earth	UAV remote sensing	Raster data	RGB image, multispectral image	Forest landscape texture, single tree morphology, vegetation index (NDVI and other derived indicators)
Field inventory	devices used for measuring dimensions etc.	Structured, semi-structured	Tree growth (height, diameter at breast height, age), stand structure (tree species composition, density), soil data (type, fertility)	Individual/community structure of trees, basic soil properties
Positioning and terrain	GPS,Beidou and other positioning technologies	Vector data	Longitude and latitude, altitude	Spatial location, topography and landforms
Dynamic monitoring	WSN	Structured, semi-structured	Environmental factors (temperature and humidity, wind speed), soil parameters (moisture, nutrients), tree physiology (sap flow)	Dynamics of forest microenvironment and physiological activities of trees
Meteorological data	Meteorological department, Weather station	Structured, emi-structured	Precipitation, temperature, humidity, solar radiation	Regional climate factors
Multi-Platform Convergence	Satellite, UAV, Sensor, Intelligent terminal	Multi type fusion data	Multi platform collaborative collection of optical, radar, physiological, and environmental data	Macro wide area, meso high resolution, and micro precise data integration

### 2.3 Multimodal data preprocessing methods

As a “bridge” for fusion, preprocessing ensures data quality through data cleaning, establishes correlations via spatiotemporal registration, and uncovers value by means of feature extraction. Ultimately, it offers reliable input for the intelligent analysis of models. Its main steps include three stages: data cleaning, spatiotemporal registration, and feature extraction and dimensionality reduction (Bhattarai et al., 2021). **Table 3** presents the technical methods corresponding to each core link of multi-source data preprocessing (data cleaning, spatiotemporal registration, feature extraction and dimensionality reduction) and the adapted data types.

**Table 3:** Core steps of multi-source data preprocessing.

Preprocessing steps	Technical method	Data type
Data cleaning	Cloud mask (Fmask, Sen2Cor)	Remote sensing (optical/SAR/LiDAR), Internet of Things
	Speckle noise suppression (Lee filtering, Kuan filtering)	
	Outlier detection (IQR/Z-score, threshold ±3σ, linear interpolation correction)	
Spatiotemporal registration	Geometric correction (rational function model RFM, ground control point GCP)	Remote sensing-ground-Internet of Things-location data
	Coordinate transformation (CGCS2000/UTM projection)	
	Time synchronization (timestamp alignment)	
Feature extraction and dimensionality reduction	Spectral characteristics (NDVI/EVI/PRI)	Remote sensing, survey, and IoT data
	3D features (CHM/DHM)	
	Statistical features (mean/variance/texture GLCM)	
	Dimensionality reduction(PCA/T-SNE)	

#### 2.3.1 Data cleaning

As a key link in ensuring data quality (Josi et al., 2025), data cleaning targets forestry multi-source data (remote sensing, Internet of Things, survey data, etc.). For remote sensing data such as optical images and SAR data, methods including cloud masking (e.g., Fmask, Sen2Cor, which combine spectral thresholds and spatiotemporal interpolation to improve cloud detection accuracy and ensure the reliability of subsequent data extraction), speckle noise suppression (Lee filtering, Kuan filtering, which smooth noise in SAR/LiDAR data while preserving edge information to optimize data quality), and outlier detection and repair (IQR/Z-score to identify outliers, and linear or spatial interpolation to fill in missing values, suitable for error repair of IoT sensor data and survey data) are adopted (Chaves et al., 2021). For ground plot survey data, logical verification (matching of tree species and site conditions, correlation of tree growth parameters, reasonable correlation of stand structure, etc.) and spatial matching (Buffer analysis to ensure spatial consistency with remote sensing images) are used to comprehensively improve the quality of forestry multi-source data.

#### 2.3.2 Spatiotemporal registration

“Spatial-temporal registration” is a key preprocessing step for realizing multi-source data fusion in forestry, covering three core components: geometric correction, coordinate transformation, and temporal synchronization (Atehortúa et al., 2020). For geometric correction, the Rational Function Model (RFM) combined with Ground Control Points (GCPs) is adopted to correct remote sensing images to real geographic coordinates, with the error controlled within 1 pixel, and this method can also be extended to the registration of LiDAR point clouds and optical images; coordinate transformation serves to unify the coordinate systems of multi-source data, avoiding “coordinate system conflicts” in spatial analysis; temporal synchronization aligns data to a unified time scale by extracting timestamps and performing linear interpolation or resampling, and a three-level geometric registration system (coarse registration, fine registration, and sub-pixel registration) and a dual temporal synchronization mechanism (hard synchronization, soft synchronization) are established to fully ensure the spatial-temporal consistency of data.

#### 2.3.3 Feature extraction and dimensionality reduction

“Feature Extraction and Dimensionality Reduction” is a critical link in exploiting the value of multi-source forestry data(S. Wang et al., 2023b), which targets remote sensing, survey, and Internet of Things (IoT) data to extract and optimize multi-dimensional features; spectral features include the Normalized Difference Vegetation Index (NDVI, which reflects vegetation coverage), Enhanced Vegetation Index (EVI, which resists saturation in high-coverage areas), and Photochemical Reflectance Index (PRI, which enables early detection of plant stress), all of which accurately characterize the physiological status of vegetation; three-dimensional features consist of the Canopy Height Model (CHM, generated from LiDAR point clouds) and Digital Height Model (DHM, which reflects topographic elevation and assists in explaining differences in vegetation distribution); statistical features comprise mean/variance (reflecting the average vitality of vegetation) and Gray-Level Co-Occurrence Matrix (GLCM)-based texture features (which calculate parameters such as contrast and support tree species classification); features of different dimensions first require dimensionality reduction, with main techniques including Principal Component Analysis (PCA, a linear dimensionality reduction method that improves computational efficiency) and t-Distributed Stochastic Neighbor Embedding (t-SNE, a non-linear dimensionality reduction method that preserves local features), and these processed features offer effective input for intelligent forestry analysis.

### 2.4 Multimodal data fusion strategies

multimodal data fusion methods mainly include two categories: traditional machine learning-based fusion and deep learning-based fusion (Chehreh et al., 2023). In traditional methods, feature-level fusion concatenates features from different modalities (such as spectral vegetation indices and LiDAR structural parameters) into a unified vector as model input, while decision-level fusion achieves integration by weighted averaging the prediction results of independent models. In deep learning methods, early fusion merges multi-modal data (such as RGB images and LiDAR point cloud projections) into a unified input tensor during the preprocessing stage; late fusion extracts modal features separately via a two-stream network and concatenates them before the output layer; hybrid fusion combines the two aforementioned approaches to realize cross-modal interaction at different network layers. Among these, deep learning methods can automatically learn modal weights through mechanisms like attention, thereby improving fusion performance in complex scenarios.

#### 2.4.1 Traditional machine learning fusion

A single model often has limitations and is difficult to meet the requirements of complex tasks. Traditional machine learning fusion strategies can improve the prediction accuracy, robustness, and generalization ability of the model by combining the prediction results of multiple models (Fathololoumi et al., 2022).

Feature-level fusion is one of the fusion strategies in traditional machine learning; it targets multi-modal forestry data (such as multi-spectral images, LiDAR point clouds, and ground survey data), extracts features with clear physical meanings (e.g., multi-spectral vegetation indices including NDVI and EVI that reflect vegetation physiology, LiDAR point cloud statistics that characterize three-dimensional structures, and phenological features derived from multi-temporal multi-spectral data), splices these features into a unified feature vector based on information complementarity, and inputs the vector into traditional models (such as random forests and SVM). It includes spectrum-structure feature fusion (which combines vegetation indices with point cloud statistics) and temporal-spectrum fusion (which combines multi-temporal phenological features with single-temporal structural features). Its advantages are clear physical meanings, strong model interpretability, and adaptability to small and medium-sized datasets (<1000 samples), while its limitations are that it relies on manual feature engineering and is difficult to capture complex non-linear relationships (e.g., the coupling effect between spectrum and structure in dense forests).

Decision-level fusion is a fusion strategy applied in traditional machine learning for multi-modal forestry data. It targets multi-modal forestry data (such as hyperspectral data and LiDAR point clouds), enabling different types of modal data to be independently input into their respective adaptive models (e.g., SVM for processing hyperspectral data and k-NN for processing LiDAR point clouds), and then integrates the prediction results through methods like voting and weighted average. Its main advantages include strong independence between models, high expandability, and adaptability to forestry scenarios with significant differences in data quality (e.g., sparse LiDAR point clouds in some regions); while its limitations are that it ignores the underlying correlations between modalities (such as the micro-coupling between spectrum and structure) and the improvement of accuracy depends on the degree of difference between models. In integrating multi-source data from Sentinel-1 and Sentinel-2 (Lechner et al., 2022), took the Wienerwald Biosphere Reserve in Austria, Central Europe, as the study area, adopted the random forest classifier, and explored the classification effects of Sentinel-1 (microwave data) and Sentinel-2 (optical data) on 12 tree species (7 deciduous species and 5 coniferous species) when used individually and in combination.

#### 2.4.2 Deep learning fusion

**Figure 2:**
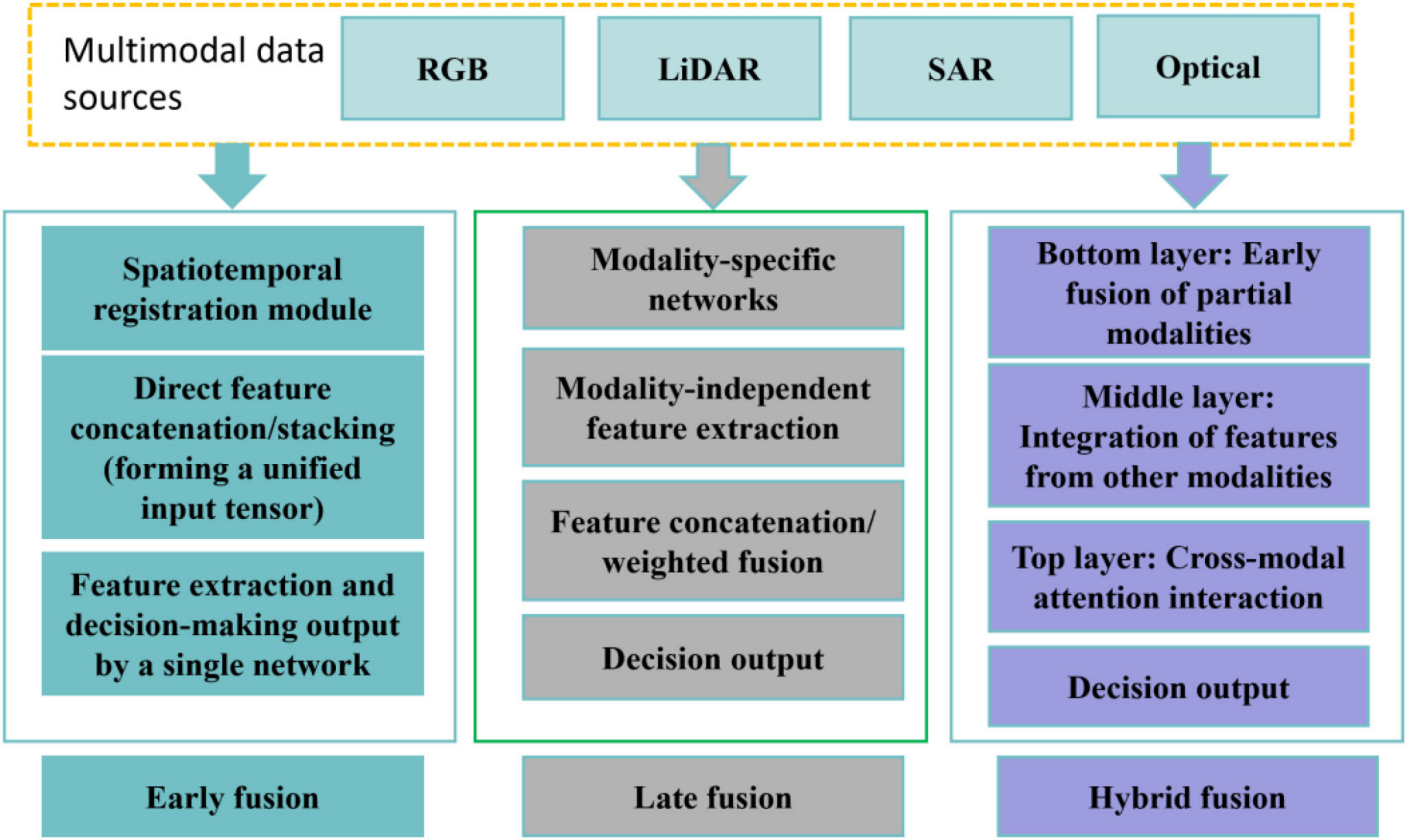
Multimodal data fusion methods.

Deep learning breaks the dependence of traditional methods on manually designed features by virtue of adaptive feature extraction and fusion mechanisms. Among them, homogeneous fusion refers to the fusion of multi-source data with the same type and consistent data structure. For example, in the fusion of the same type of data such as optical remote sensing images, the residual connection of CNN is used to strengthen the coherence of feature transmission, and the generative effect is optimized in combination with the adversarial training of GAN, which can increase the spatial detail retention of pansharpening by 10% - 15%, making details such as ground object edges more clearly displayed after the fusion of high-resolution panchromatic images and multispectral images. Heterogeneous fusion is the fusion of multi-source data with different types and different data structures. For example, when fusing heterogeneous data such as SAR radar data, optical remote sensing images, and ground survey text data, dual-branch networks are used to process and interact with different types of inputs respectively, and combined with cross-attention modules to focus on key associated information, the OA (Overall Accuracy) of HS-LiDAR (Hyperspectral - Light Detection and Ranging) classification can be increased from 80.39% of single modality to 89.60%, fully demonstrating its excellent representation ability for multi-source heterogeneous data(J. Li et al., 2022). **Figure 2** shows the multimodal data fusion methods, including three technical paths: early fusion, late fusion, and hybrid fusion, which are based on multimodal data sources.

Early fusion integrates multi-modal data at the data input layer to form a unified tensor (such as a 2D grid or multi-channel matrix), and leverages networks like CNN for end-to-end feature learning. It includes image-point cloud fusion (converting LiDAR point clouds into a Canopy Height Model and superimposing it with RGB image channels) and multi-channel feature concatenation (merging hyperspectral data with RGB channels and using 3D convolution to extract cross-modal spectral features, which compensates for the low spatial resolution of hyperspectral data). Its advantages are high computational efficiency (due to end-to-end training) and adaptability to scenarios where modalities have good spatial registration (e.g., data collected synchronously by UAVs); its limitations are extremely high requirements for spatiotemporal registration (fusion performance degrades when the error exceeds 1 pixel) and vulnerability to the “curse of dimensionality” caused by modal differences (e.g., dimensional expansion in the fusion of hyperspectral and RGB data.

Late fusion involves extracting features from different modal data through independent networks (adapted to the characteristics of each modality, e.g., CNN for processing images and PointNet/PointNet++ for processing point clouds), followed by concatenation and fusion before the output layer. This method has the advantages of flexibly adapting to modal differences, being capable of learning dynamic weights, and being suitable for complex forestry scenarios such as cloudy conditions and dense forests; its limitations include a large number of parameters and high training costs. When utilizing multi-modal data (Ahlswede et al., 2023), avoided the issue of spatial information distortion caused by early fusion by processing the data from each sensor independently.

Hybrid fusion is a multi-modal forestry data fusion strategy that combines early fusion and late fusion, enabling cross-modal interaction at different network levels, with a typical model of “hierarchical fusion + cross-modal generation”. Hierarchical fusion involves fusing spectral data at the bottom layer (convolutional layer) to capture subtle spectral differences (e.g., red edge shift caused by diseases and pests) and introducing structural features (e.g., forest layer height distribution) at the upper layer (pooling layer/fully connected layer), forming a “bottom-up” cross-modal information flow; the cross-modal generation model uses GAN to generate virtual multi-modal data (e.g., generating hyperspectral virtual images based on LiDAR) to enhance the diversity of training samples. Its advantages include balancing low-level details (e.g., individual tree spectral anomalies) and high-level semantics (e.g., forest stand structure types), as well as improving performance in complex scenarios such as cross-seasonal periods and cloudy/rainy areas; its limitations are complex network design (requiring customized hierarchical interaction structures), high parameter tuning difficulty (needing fine optimization of cross-modal weights, etc.), and high technical thresholds. Meanwhile, hybrid fusion covers homogeneous fusion (processing similar types of data to improve spatiotemporal/spectral resolution) and heterogeneous fusion (processing data with different imaging mechanisms to achieve information complementarity, e.g., HS-LiDAR fusion for estimating forest biomass and SAR-optical fusion for monitoring crops in cloudy areas).

**Figure 3:**
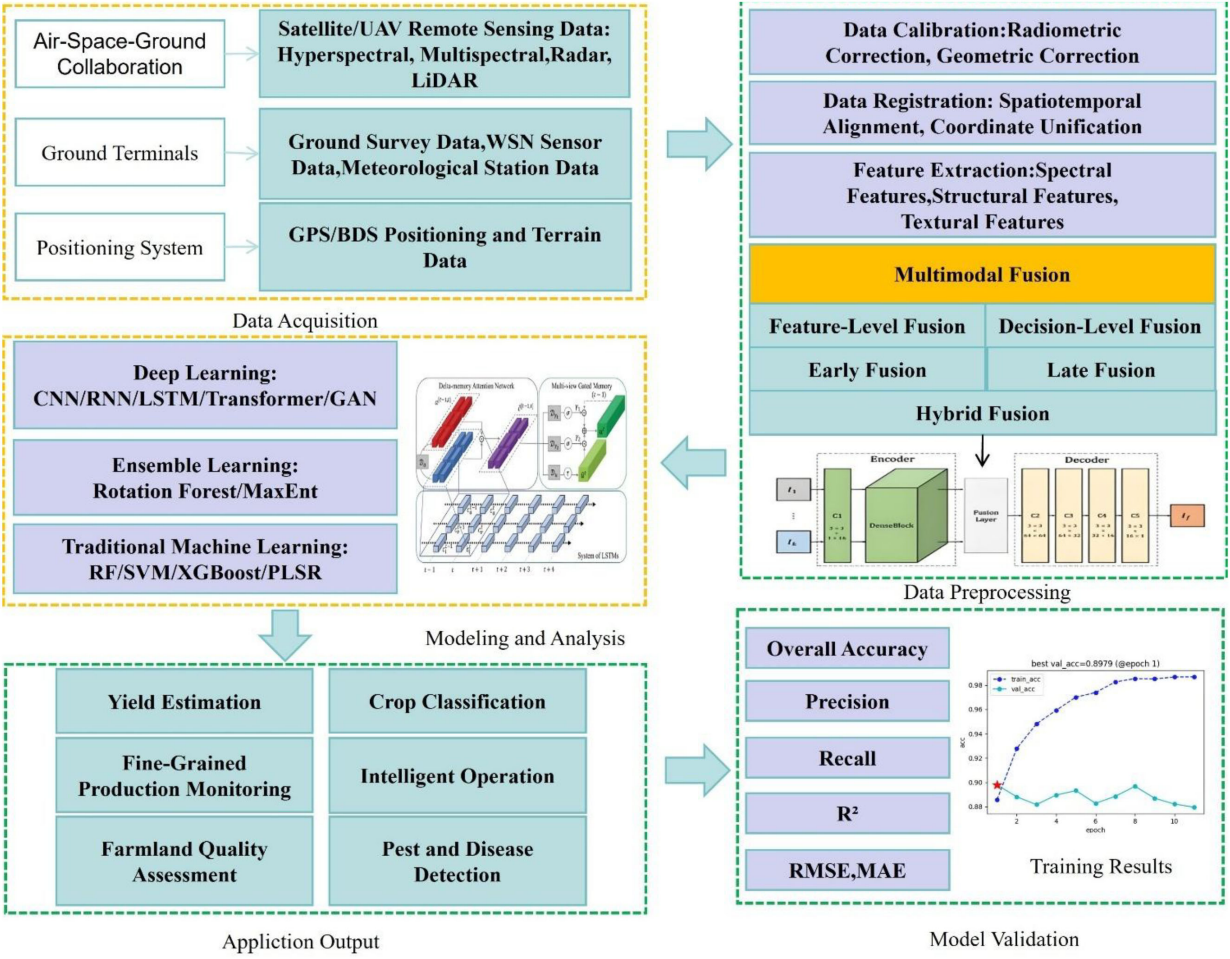
Technical roadmap of multimodal fusion.

Feature-level fusion and decision-level fusion are widely applied in small and medium-sized datasets, relying on the physical interpretability of manually designed features. Deep learning fusion is a trend: late fusion and hybrid fusion perform better in large-scale datasets (>10,000 samples), and are especially suitable for complex scenarios (such as dense forests and cross-season monitoring). In addition, innovations are constantly being made in multimodal learning frameworks. To address the bottleneck of single-modal deep learning in remote sensing image classification in complex scenarios (Hong et al., 2021), proposes a general multimodal deep learning (MDL) framework, focusing on “fusion content, location, and method”, and designs five architectures: early, middle, late, encoder-decoder (En-De), and cross-fusion, integrating pixel-level fully connected networks (FC-Nets) and spatial-spectral convolutional neural networks (CNNs). Experiments on HS-LiDAR and MS-SAR datasets show that the cross-validation accuracy reaches 98.6% and the test set validation reaches 91.2%. Among them, the cross-fusion strategy performs prominently in cross-modal learning (CML), effectively improving classification robustness and providing a promotable multimodal fusion scheme for accurate classification of remote sensing images. **Figure 3** presents a technical roadmap for multimodal fusion, covering the complete process from data acquisition methods such as air-space-ground collaboration, through data preprocessing, multimodal fusion, and modeling analysis, to application output and model validation.

## 3 Application scenarios of multimodal data fusion in forestry monitoring

In forestry monitoring, multimodal data fusion technology has achieved remarkable progress in multiple scenarios such as tree species classification, land resource monitoring, forest structural parameter estimation, disaster monitoring, and tree health assessment by integrating multi-source heterogeneous data and deep learning algorithms.

### 3.1 Tree species classification

**Figure 4:**
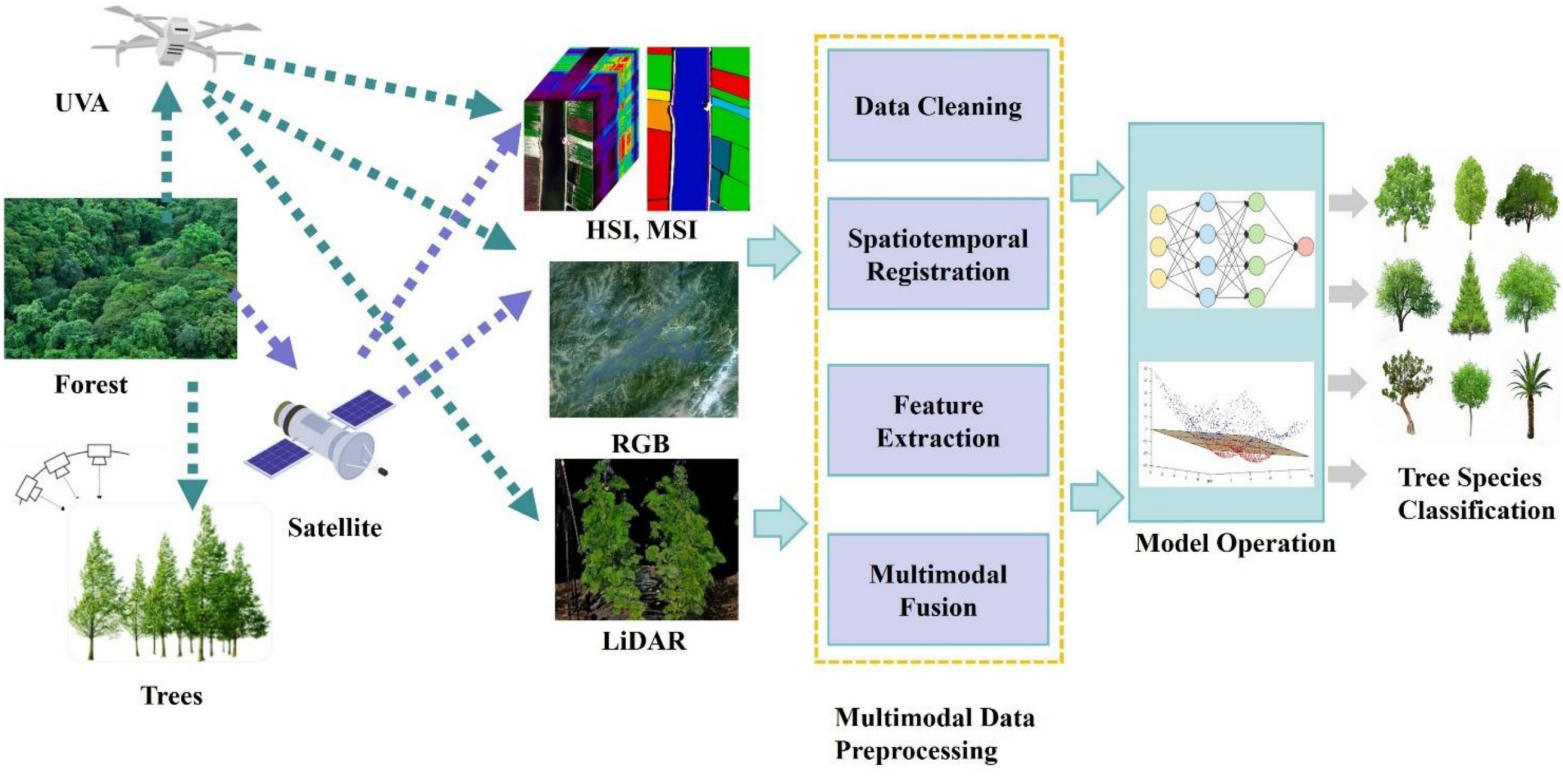
Schematic diagram of tree species classification application.

Tree species classification is the core of precise management of forest resources. Traditional morphological classification relies on expert experience. Although it is suitable for small-scale precise identification, it has the bottlenecks of low efficiency and difficulty in large-scale application (Fassnacht et al., 2016). pointed out several issues in tree species classification at that time: there were very few cases of complex fusion of heterogeneous data such as spectral images and LiDAR; traditional classification algorithms struggled to adapt to the high-dimensional characteristics of multimodal data; and most studies lacked spatially independent validation strategies and attention to cost-effectiveness. The tree species classification methods represented by machine learning or deep learning have continuously improved in prediction accuracy and efficiency, and have become the current mainstream of tree species classification. Based on the applications in tree species classification in recent years (Luo et al., 2023; L. Zhong et al., 2024), hyperspectral (HSI), LiDAR and RGB are the most used in single-modal data, and multimodal data fusion is mainly HSI+LiDAR; CNN has become the mainstream classification method, and models such as ResNet have an accuracy of over 90% in small-scale classification. In addition, multimodal data is also widely used in urban tree species classification(F. Fang et al., 2020; C. Zhang et al., 2021), which is of great significance to urban species monitoring and planning. **Figure 4** shows the process of tree species classification application, that is, multi-source data are acquired by satellites, UAVs, etc., and after multimodal data preprocessing, tree species classification is realized by model operation.

#### 3.1.1 Evolution and bottlenecks of single-modal classification technology

##### 3.1.1.1 Traditional classification methods and breakthroughs in remote sensing technology

Single-modal classification centers on remote sensing technology and is prone to interference from external factors; numerous studies have gradually improved its accuracy through innovations in data and algorithms (Marconi et al., 2022). established a cross-scale classification model for single spectral remote sensing, confirming that cross-scale models can improve accuracy; (Axelsson et al., 2021) applied Bayesian sequential inference to Sentinel-2 multi-temporal images for the first time, dynamically updating category likelihood probabilities, solving the problem of cloud occlusion; (Xi et al., 2021) compared deep learning algorithms such as Conv1D and LSTM with RF and SVM algorithms, and found that the Conv1D model achieved an OA of 84.19% in multi-temporal Sentinel-2 classification; (T. Ma et al., 2021) collected diffuse reflection spectra of 15 kinds of wood, constructed a classification model by combining support vector machine (SVM) after dimensionality reduction through principal component analysis (PCA), with a cross-validation accuracy of 98.6%. However, a single remote sensing data source (such as optical or microwave) is difficult to balance spectral details and all-weather acquisition capabilities, and traditional methods have insufficient classification accuracy due to complex terrain and differences in vegetation phenology (Lechner et al., 2022).

Extensive research has also been carried out in the field of UAV multispectral (Guo et al., 2022). used 0.01m resolution UAV multispectral images, combined with object-oriented segmentation and random forest. Among them, providing an efficient method for urban tree classification; (Veras et al., 2022) obtained multi-season 4cm resolution RGB images through low-cost UAS, combined with the DeepLabv3+ model, which increased the classification accuracy of tropical forests by 21.1% to 90.5%; (Chadwick et al., 2022) took the regenerated coniferous forest in Alberta, Canada as the research scene, used RGB (3cm) and NIR (5cm) images obtained by UAV, outlined the tree crown through Mask R-CNN, and realized logistic regression classification; (Abdollahnejad and Panagiotidis, 2020) used UAVs to collect multispectral data of tree species, extracted their vegetation index features and texture features, and combined with SVM to realize tree species classification and health assessment of mixed forests.

##### 3.1.1.2 3D structure and single-modal exploration of deep learning

LiDAR plays a significant role in scenarios such as tree species classification due to its ability to capture spatial structure and intensity information (Cetin and Yastikli, 2022). combines 3D LiDAR point clouds with algorithms like SVM and RF, improving the accuracy of urban tree species classification through the fusion of spatial and intensity features, thus breaking through the spectral limitations of optical remote sensing. (J. Zhou et al., 2023) uses 4cm resolution UAV images and the BlendMask algorithm, achieving OA = 92.14% in coniferous forest classification. With the development of deep learning, its advantages in tasks such as tree species classification have gradually become apparent (Gahrouei et al., 2024). combines deep learning methods such as DenseNet and ResNet with algorithms like RF and SVM to classify 9 tree species, with DenseNet performing the best (OA = 78%) (Harmon et al., 2023). combines CNN with the DeepCTRL framework, integrating domain knowledge such as crown height and altitude, increasing the F1 score of rare tree species by 8.3 points (Beloiu et al., 2023). uses 10cm resolution aerial RGB images and the Faster R-CNN model to detect and identify individual tree crowns of 4 tree species including Norway spruce. The model’s average F1 score for single species is 0.76. In forestry monitoring, multimodal data fusion technology has achieved remarkable progress in multiple scenarios such as tree species classification, land resource monitoring, forest structural parameter estimation, disaster monitoring, and tree health assessment by integrating multi-source heterogeneous data and deep learning algorithms (Fassnacht et al., 2016).

#### 3.1.2 Multimodal data fusion

Multimodal fusion focuses on optics, radar, and topographic factors, constructing a technical chain of “data registration - feature fusion - model integration”, and carries out explorations from data integration, registration optimization, and scenario-based applications. The core value of data fusion lies in breaking through the limitations of single data and improving classification effects through the complementarity of multiple types of data, which is a key link to verify algorithm adaptability and data gain. (Xin Chen and Sun, 2024) fused Sentinel-1/2, NFRI data and topographic factors, and adopted random forest and gradient tree boosting to achieve the extraction of dominant species in subtropical forests with OA = 83.6% (Zheng et al., 2023). integrated Sentinel-1/2, terrain, temperature, and precipitation data, compared RF/SVM/XGBoost, confirmed that the full data combination + RF algorithm achieved OA = 77.98%, and revealed the key contributions of environmental factors such as rainy season precipitation and altitude. Data registration accuracy directly affects the quality of feature fusion and is a basic supporting link of multimodal fusion. It is necessary to optimize matching methods in a targeted manner to solve the problem of spatial coordination of heterogeneous data. (Y. Xu et al., 2023) proposed a tree-oriented matching method based on maximum crown overlap, which increased the pairing rate of single trees between aerial photos and LiDAR from 91.13% to 100%, and the matching accuracy NIoU from 0.692 ± 0.175 to 0.861 ± 0.152, laying a spatial foundation for feature fusion. Forest protection scenarios have an urgent demand for multimodal fusion, which needs to combine actual threat problems, help resource management decisions through technology implementation, and realize the transformation of research value (Potter et al., 2019). took the threat of insects and diseases to North American forest tree species as the scenario, developed the Project CAPTURE framework, integrated data on threat severity, sensitivity, and adaptability of 419 native tree species, and used K-means clustering and expert weight algorithms to divide the tree species into 11 vulnerability classes, finding that 15 of the most vulnerable tree species need urgent protection. The research offers a framework for the scientific allocation of protection resources, emphasizing the combination of expert opinions and quantitative analysis to assist in the management of forest genetic resources.

Traditional machine learning fusion has achieved certain results in the utilization of multimodal data. However, in the face of higher requirements for accuracy and efficiency in complex scenarios, deep learning has become a breakthrough direction relying on the advantages of automated feature extraction and cross-modal interaction, with explorations carried out from end-to-end frameworks, weakly supervised learning, and multi-scenario model applications. End-to-end multimodal frameworks focus on building integrated models, integrating data features of different dimensions, and exploring complementary values. (B. Liu et al., 2023) developed the TSCMDL framework, by fusing UAV-borne LiDAR (3D) and RGB (2D) features, the achieved classification accuracy was 4.02% higher than that of the single LiDAR modality (Vahrenhold et al., 2025). proposed MMTSCNet, which fuses point cloud, depth image and other data through a four-branch structure, combined with dynamic modal scaling, making the OA of multi-source single-tree LiDAR classification close to 97%. Weakly supervised learning and dataset innovation focus on reducing annotation costs, expanding data fusion types, and further releasing multimodal potential (Amin et al., 2024). developed a weakly supervised model, which uses visible/near-infrared images + topographic data, combined with ResNet50 and pseudo-label technology. In the classification of 9 tree species in Cyprus, the OA is 90%, and the annotation cost is reduced by more than 50% (Aburaed et al., 2023). confirmed that after fusing HSI and MSI with the CNMF method, the total accuracy of tree species classification reaches 89.2%, which is 3.1% higher than that of single HSI. The combination of multi-source images and algorithm adaptation is also a research focus. The combination of different data sources and models verifies the effect in scenarios such as single-tree analysis. (Xianggang Chen et al., 2023) uses UAV RGB and SuperView-1 multispectral images, combines the object-oriented MRS algorithm to segment single trees, extracts texture and spectral features, and adopts random forest as well as deep learning networks such as MobileNetV2, ResNet34, and DenseNet121 for classification. The results show that stand density has little impact on segmentation, the classification accuracy of deep learning networks is higher than that of random forest, and DenseNet121 performs the best.

In addition to the above directions, fusion models for specific data types (such as LiDAR and multispectral) continue to innovate, expanding the application scenarios of forest monitoring (Briechle et al., 2021). used airborne LiDAR and multispectral images to propose the Silvi-Net dual CNN framework, which renders LiDAR point clouds into multi-view images and fuses MS image patches, extracts features through ResNet-18, and then classifies them by MLP. The dataset contains LiDAR and MS data of forest stands with different densities in two locations. Experiments show that the overall accuracy of the model reaches 96.1% and 91.5%, which is significantly higher than that of PointNet++, proving that multi-source data fusion can efficiently classify tree species and dead trees, providing a new scheme for forest monitoring.

In the research process of multimodal fusion, from the verification of high-precision classification in complex scenarios, to the exploration of cross-modal information complementary mechanisms, and then to the breakthrough of large-scale classification problems, the application value and advantages of multimodal fusion have been gradually demonstrated. Through cases in different scenarios, the understanding of fusion effects has been deepened. Literatur (Qin et al., 2022) fused UAV LiDAR, hyperspectral and RGB, achieving 18 tree species classification in Shenzhen subtropical broad-leaved forest with OA = 91.8%, which was significantly higher than that of single data (Cao et al., 2021). fused UAV-borne hyperspectral data and LiDAR data, and employed the Rotation Forest algorithm. This approach enabled the mangrove classification accuracy to reach 97.22%, demonstrating the significance of cross-modal information complementarity. (H. Zhong et al., 2022) fused hyperspectral and LiDAR in the coniferous-broadleaved mixed forest of Northeast China, with the classification accuracy reaching 89.20%, which was better than that of single hyperspectral (86.08%) and LiDAR (76.42%), verifying the complementarity of spectral-structural features. (Y. Li et al., 2022) proposed ACE R-CNN, which fused RGB and CHM generated by LiDAR, and through the optimization of the attention module, the precision of single tree recognition exceeded 0.9. Large-scale tree species classification is more challenging due to data heterogeneity and large spatial span, so it is necessary to explore adaptive technical frameworks. Multimodal fusion offers solutions from the perspectives of regionalization and model integration. (P. Fang et al., 2023) addressed the challenge of large-scale tree species classification by fusing Sentinel-2 imagery, SRTM DEM data, and WorldClim data. It developed a framework of “regional division + multi-source feature fusion + model integration”; by adopting this integrated model, the overall classification accuracy reached 72.18%, which was significantly higher than that of single models.

Most studies (e.g (Cao et al., 2021; Xin Chen and Sun, 2024)) only verify the effectiveness of a single strategy in specific scenarios, lacking horizontal comparisons of multiple strategies under the same dataset (e.g., failing to simultaneously test the performance differences between traditional machine learning (ML) and deep learning in the subtropical broad-leaved forest scenario). This leads to the conclusion of an “optimal strategy” lacking universal support. Additionally, some studies (e.g., (P. Fang et al., 2023)) overemphasize the accuracy improvement brought by model integration, yet fail to discuss practical application bottlenecks such as “the subjectivity of regional division thresholds” and “the computational cost of multi-model parallelism,” making it difficult to guide engineering implementation. For extreme scenarios like “dense closed forests” and “cloudy and rainy areas,” existing fusion strategies (e.g (Qin et al., 2022; H. Zhong et al., 2022)) still rely primarily on optical data. The penetrability advantage of Synthetic Aperture Radar (SAR) data has not been fully utilized, and further exploration of the “SAR-optical-LiDAR” three-modal deep fusion architecture is required.

#### 3.1.3 Tree species classification based on multimodal UAV data

With its high-resolution capability and multi-sensor integration capacity, unmanned aerial vehicles (UAVs) have become the core carrier for multi-modal classification. In terms of data acquisition, the sub-meter to centimeter-level resolution breaks through the bottlenecks of satellite data, enabling individual tree-scale analysis. Additionally, UAVs can be equipped with sensors such as LiDAR and hyperspectral sensors to synchronously acquire multi-dimensional information, including spectral data (e.g., NDVI), 3D structural data (e.g., CHM), and physio-chemical data (e.g., hyperspectral fingerprints). During fusion processing, after preprocessing steps like spatiotemporal registration and feature dimensionality reduction (e.g., PCA/t-SNE), multi-modal algorithms (such as feature concatenation in traditional machine learning, and two-stream networks or attention mechanisms in deep learning) are integrated to achieve the complementarity between spectral and structural data. Specifically, LiDAR addresses the problem of spectral occlusion in closed-canopy forests, while spectral data enhances the identification of spectral differences between species—both contributing to improved classification accuracy. Meanwhile, this approach is compatible with small-sample learning and interpretability analysis, enhancing the robustness of the model and its association with ecological mechanisms.

In application practice (Quan et al., 2023), took the natural secondary forest in Mao’er Mountain, Northeast China as the research scenario, using UAV LiDAR and hyperspectral data, through mixed feature selection and random forest algorithm, to classify 11 common tree species. The accuracy after fusion reached 75.7%, which was better than that of single data; (J. Zhou et al., 2023) used 4cm resolution images and the BlendMask algorithm, and the producer accuracy of coniferous trees reached 0.91-0.95; (H. Zhong et al., 2022) fused hyperspectral and LiDAR, with the total accuracy of single-tree segmentation being 84.62% and the accuracy of tree species identification being 89.20%; (B. Wang et al., 2023) designed 12 schemes for various tree species in Mao’er Mountain Forest Farm using UAV LiDAR and hyperspectral data, combined with algorithms such as random forest, confirming that the classification accuracy of multi-source data (up to 79.91%) is better than that of a single data source; (Y. Li et al., 2022) proposed the ACE R-CNN algorithm, which fuses UAV RGB images with CHM generated by LiDAR, and realizes single tree species identification after optimization, with precision and other indicators exceeding 0.9, contributing to forest resource management. However, there are still problems in the current forest tree species classification based on remote sensing images: the lack of benchmark datasets, most studies are limited to small areas, random forests are prone to overfitting, a single remote sensing data source is difficult to balance spectral details and all-weather acquisition capabilities, and complex terrain and differences in vegetation phenology also lead to insufficient accuracy of traditional methods. Although multimodal data fusion has formed a technical chain of “data registration - feature fusion - model optimization”, improving classification accuracy and efficiency, it is still necessary to conduct in-depth exploration in aspects such as cross-modal deep learning architectures to promote the application of technology in global forest resource refinement management.

#### 3.1.4 Future trends and technological breakthroughs

Forest tree species classification has achieved a key transformation from single-modal traditional methods to multi-modal deep learning paradigms (Bhattarai et al., 2021), and the UAV combination of HSI + LiDAR + RGB has become the core solution for small-scale high-precision classification. This transformation stems from the significant advantages of multimodal fusion: through the complementarity of “optics-radar-environment” multi-dimensional information, optical data (such as RGB cameras) offer low-cost (Jayathunga et al., 2023), easily accessible visible light spectral information, radar data (such as LiDAR) realize high-precision three-dimensional structure reconstruction of tree height, crown width, etc., and then combined with topographic factors such as altitude and slope, together with deep learning automated feature extraction, the classification accuracy is 10%-20% higher than that of single modality. However, current application research has many bottlenecks, including low automation of data annotation (single-tree level annotation relies on time-consuming field surveys, few public datasets, and low proportion of weakly supervised learning applications), poor model generalization (decreased cross-regional accuracy, lack of domain adaptation mechanisms), insufficient utilization of spatiotemporal data (few studies on fusing multi-temporal data, failure to fully explore phenological trajectories), and difficulty in adapting to edge computing (complex models cause inference delays on the UAV side, making real-time monitoring impossible). Future research can focus on cross-modal deep learning architectures, weakly supervised learning, application of Transformer technology(L. Zhong et al., 2024) and edge computing applications, etc., to promote the implementation of technology in global forest resource refinement management. Specifically, spatiotemporal sharpening algorithms can be developed (such as fusing HSI + SAR time-series data to capture phenological dynamics), combined with weakly supervised + small-sample learning (using pseudo-labels and meta-learning to ensure accuracy in few-sample scenarios), design lightweight models to achieve real-time classification on the UAV side, the adoption of Vision Transformer (ViT) improves the performance of individual tree segmentation. Additionally, the development of globally shared datasets like TreeSatAI advances research on domain adaptation, facilitating the shift from “local validation” to “global-scale” accurate classification (Prodromou et al., 2024), contributing to global forest resource refinement management. At the same time, deep learning models are superior to traditional machine models in accuracy (Choi et al., 2022); for example, (T. He et al., 2023) applied ResNet and DenseNet to tree species classification and achieved good results, providing a practical basis for algorithm innovation. **Table 4** summarizes the research progress in tree species classification in different years, including the data sources, model algorithms, application fields, and achieved effects.

**Table 4:** Research progress in tree species classification.

Literature number	Data source	Model algorithm	Application area	Effect	Year
(Xin Chen and Sun, 2024)	Remote sensing data, NFRI data, Terrain data	RF, GTB	Tree species classification	Overall accuracy OA 97.4%	2023
(Y. Xu et al., 2023)	Aerial image, LiDAR	Tree oriented matching	Tree Space Matching	The average pairing rate has increased from 91.13% to 100%	2023
(B. Liu et al., 2023)	LiDAR, RGB	TSCMDL	Tree species classification	The TSCMDL classification accuracy reaches 98.52%	2023
(Vahrenhold et al., 2025)	LiDAR, FWF	MMTSCNet	Tree species classification	The overall accuracy (OA) is nearly 97%,	2025
(Gaydon and Roche, 2024)	ALS point cloud, aerial imagery	RandLA-Net	Tree species classification	The overall accuracy (OA) on the test set is 80.3%	2024
(Amin et al., 2024)	RGB, satellite image, terrain features	ResNet50	Tree species classification	Reached 90% after fusing terrain features	2024
(Zheng et al., 2023)	Sentinel-1, Sentinel-2, environmental data	RF, SVM, XGBoost	Tree species classification	The overall accuracy (OA) reaches 77.98%	2023
(Qin et al., 2022)	LiDAR, hyperspectral RGB	WST-NCut	Tree species classification	The tree species classification accuracy rate reaches 91.8%	2022
(Quan et al., 2023)	LiDAR, hyperspectral imaging	RF	Tree species classification	Overall accuracy (OA) 75.7%, Kappa coefficient 0.753	2022
(Cao et al., 2021)	Hyperspectral,LiDAR data	RoF	Tree species classification	The overall accuracy rate reaches 97.22%	2021
(Xianggang Chen et al., 2023)	RGB, multispectral image, field measurement data	RF, MobileNetV2, ResNet34, DenseNet121	Tree species classification	The deep learning network achieves the best performance	2023
(P. Fang et al., 2023)	Sentinel-2 image, SRTM DEM WorldClim	MaxEnt, RF, SVM, XGBoost fusion	Tree species classification	The overall accuracy of the fusion model is 72.18%	2023
(Briechle et al., 2021)	Multi spectral images, LiDAR	Silvi Net Dual CNN Framework	Tree species classification	The fusion of LiDAR and MS improves ChEZ OA by 2.5%	2021
(Lechner et al., 2022)	Sentinel-1 image, Sentinel-2 image	RF	Tree species classification	S1 fuses S2 multi temporal data to make up for the deficiency of single modal data	2022
(B. Wang et al., 2023)	LiDAR, hyperspectral data	RF, SVM, BP	Tree species classification	The highest classification accuracy is 79.91%	2023
(Y. Li et al., 2022)	RGB, LiDAR	ACNet	Tree species classification	The average precision is 0.9	2022
(H. Zhong et al., 2022)	Hyperspectral data, LiDAR	SVM	Tree species classification	The total accuracy of tree species recognition reaches 89.20%	2022

### 3.2 Land resource monitoring

As core underpinnings for understanding the structure and functions of forest ecosystems, forest soil environment monitoring and land cover classification are of crucial significance to the accurate assessment of carbon cycles, systematic conservation of biodiversity, and sustainable management of forest resources. Among these two components, forest soil environment monitoring enables the quantitative analysis of forest ecosystem service values (e.g., in soil and water conservation, carbon sequestration capacity) by acquiring soil physical properties (e.g., moisture content, erosion degree) and vegetation spatial distribution characteristics, thereby providing scientific data support for formulating climate change response strategies. In contrast, land cover classification facilitates the optimization of logging plans, dynamic assessment of wildfire risks, and implementation of ecological conservation policies through the accurate identification of forest types (e.g., coniferous forests, broad-leaved forests) and their spatial patterns, further enhancing the scientific rigor and target-oriented nature of forest resource management. In terms of technical implementation, a technical framework centered on remote sensing data and integrated with statistical models or machine learning algorithms has been developed in this field. For single-modality technology applications: on the one hand, optical remote sensing (with Sentinel-2 as a typical example) can retrieve vegetation coverage and soil properties via spectral indices (e.g., NDVI), but it is significantly limited by cloud cover and vegetation canopy obstruction; on the other hand, LiDAR technology enables the extraction of vertical forest structure parameters (e.g., tree height, canopy density) using point cloud data, which is suitable for three-dimensional forest structure analysis yet highly sensitive to topographic relief. Both types of single-modality technologies have obvious application limitations. To address these bottlenecks, multimodal data fusion technology has emerged. By integrating the spectral features of optical remote sensing and the structural information of LiDAR, this technology adopts neural network models (e.g., dual-branch late fusion architecture) to automatically explore cross-modal associated features—for instance, using Convolutional Neural Networks (CNNs) to process the temporal features of images and Multi-Layer Perceptrons (MLPs) to analyze key LiDAR indicators. Ultimately, it improves the estimation accuracy of forest parameters (e.g., basal area, timber volume). Meanwhile, by introducing topographic data to correct sensor system biases (Soussi et al., 2024), the robustness of the model in complex geographical environments is further enhanced.

Based on the technical potential of multimodal fusion, many scholars have focused on its applications in the fields of forest soil monitoring and land cover classification. They integrate multi-source remote sensing data such as optical, microwave, and LiDAR with ground-measured data, and combine algorithms such as XGBR, DNN, CNN, and graph networks to promote the improvement of soil moisture prediction, soil erosion identification, and land cover classification accuracy, verifying the effectiveness of multimodal fusion in enhancing the performance of Earth observation tasks in complex geographical environments. In the field of forest soil monitoring (Nguyen et al., 2022), fused Sentinel-1/2 multispectral data, ALOS DSM, and ground soil samples from Western Australia. The XGBR-GA algorithm was used to screen 21 optimal features, achieving accurate soil moisture prediction with a performance of RMSE 0.875% and R² 0.891, providing technical support for precision agriculture; (Yin et al., 2024) was based on the RGB, multispectral, and thermal infrared images of alfalfa fields collected by UAVs and the measured soil moisture data. Through the DNN model under multimodal fusion, it achieved SMC estimation (R² = 0.72, RMSE = 4.98%), which is applicable to the irrigation management of farmland with different irrigation levels and canopy types; (Miao et al., 2024) addressed the problem that traditional models insufficiently capture the relationship between soil erosion factors and multispectral data. Using P4M UAV multispectral images and factors such as R/K/LS/C/P, it constructed the DGCS-CNN model with CBAM and GFF modules, achieving the identification of small and medium-scale soil erosion with an accuracy of 96.92%, which is a significant improvement compared to the random forest (89.64%) and RUSLE (an increase of 26.59%).

In the field of land cover classification, there are also abundant research results. (X. Du et al., 2021) proposed a graph fusion network algorithm for hyperspectral and LiDAR multi-source datasets. By constructing a multimodal graph and introducing Laplacian loss and t-SNE loss, it achieved a classification accuracy of 99.68% on the Trento dataset, providing key technologies for high-precision land cover analysis in smart cities; (X. Liu et al., 2024) designed the JoiTriNet network (including encoding-decoding level fusion and MDAFM module) for optical and SAR images, which improved the classification robustness of multi-source and single-source data on the DFC2020 (10m resolution, OA = 86.06%) and Dongying (1m resolution, OA = 94.13%) datasets; (G. Wang et al., 2025) proposed the CM²FEs algorithm to address the problem of insufficient deep fusion in multimodal classification, achieving an mIoU improvement of 1.60%–3.25% on the WHU-OPT-SAR (optical + SAR), Pohang (optical + SAR), and Berlin (three-modal) datasets with low computational complexity; (W. Ma et al., 2022) constructed the AMM-FuseNet network (channel attention + dense dilated convolution) to process multimodal remote sensing data. Compared with 6 advanced models on the Hunan, DFC2020, and Potsdam datasets, it achieved optimal performance in most indicators with low accuracy loss under small samples, enhancing the reliability of land cover mapping.

Comprehensively, multimodal technology forms significant advantages by integrating optical (such as Sentinel-2 spectrum), microwave (such as Sentinel-1), LiDAR point cloud, and ground-measured data, combined with algorithms like XGBR, DNN, and CNN. Firstly, information complementarity: for example, the fusion of LiDAR 3D structure and optical spectral features improves the estimation accuracy of soil moisture (R² reaching 0.891) and canopy height (R²=0.98); secondly, model upgrading: deep learning architectures (such as dual-branch late fusion, graph networks) automatically extract cross-modal deep features, enabling the accuracy of soil erosion identification to reach 96.92% and the accuracy of land cover classification to increase by 1.60%–3.25%; thirdly, enhanced robustness: introducing terrain data to correct sensor deviations, combined with ensemble learning (such as random forest weighting), reduces the RMSE of canopy height estimation to 0.57–4.15 meters in complex terrain areas. These fully verify the key role of multimodal fusion in improving the accuracy and reliability of Earth observation tasks, providing strong technical support for the development of forest resource monitoring and management. **Table 5** shows the research progress in land resource monitoring studies, including data sources, model algorithms, application fields, effects, and publication years involved in different literatures.

**Table 5:** Research progress in land resource monitoring.

Literature number	Data source	Model algorithm	Application area	Effect	Year
(Nguyen et al., 2022)	Sentinel-1, Sentinel-2, ALOS DSM	ML, XGBR-GA	Soil Moisture Estimation	RMSE is 0.875%, R² reaches 0.891	2022
(Yin et al., 2024)	RGB, MS, TIR	PLSR, SVM, RF, DNN	Soil Moisture Estimation	R² reaches up to 0.72 (DNN model), RMSE = 4.98%	2024
(Miao et al., 2024)	Multispectral, Erosion Factors	DGCS-CNN	Soil Erosion Classification	The accuracy reaches 96.92%, which is significantly better than models such as Random Forest (89.64%), VGGNET (91.52%), RUSLE (70.33%)	2024
(X. Du et al., 2021)	HSI, LiDAR	Graph Fusion Network	Land Cover Classification	Overall Accuracy (OA) reaches up to 99.68%	2021
(X. Liu et al., 2024)	Optical Image, SAR Image	JoiTriNet	Land Cover Classification	The overall accuracy of multimodal fusion using JoiTriNet-d exceeds 86%	2024
(G. Wang et al., 2025)	Optical Image, SAR Image	CM²FEs	Land Cover Classification	The mean Intersection over Union (mIoU) of CM²FEs on WHU-OPT-SAR and Pohang datasets is 1.60%–3.25% higher than that of existing methods	2025
(W. Ma et al., 2022)	Sentinel-1/2, DEM, TOP + DSM	AMM-FuseNet	Land Cover Mapping	The mIOU is significantly improved under the fusion of multiple modal datasets	2022

### 3.3 Forest structure parameter estimation and ecological monitoring

In the land resource monitoring system, forests are a key component. The accurate acquisition of their structural parameters (such as canopy height, biomass, and phenotype (Lou et al., 2024, 2022)) is of great significance for ecological protection and resource management, and also related to work such as forest ecological restoration assessment (Doi, 2021). The following will combine multimodal data fusion technology to elaborate on forest-related monitoring applications from the dimensions of canopy height inversion, biomass and basal area estimation, etc.

In terms of canopy height inversion, numerous studies have made progress with the help of multimodal data fusion (Xiao et al., 2025). used AAV images, Sentinel-1, and DEM data to achieve ultra-high-definition mapping of urban forest canopy height (R²=0.98 under 1-meter DEM) through the ARFCNet model, balancing spatial resolution and coverage; (Ling et al., 2025) integrated GEDI/ICESat-2 LiDAR, UAV images, and ground plot data to monitor changes in canopy height of Hainan tropical rainforests using the random forest algorithm, and found an overall upward trend from 2003 to 2023; (Goel et al., 2025) combined sparse spaceborne LiDAR (GEDI) with multi-sensor time series to construct a local canopy height model (CHM), with RMSE in multiple regions lower than that of single-sensor models; (Shufan Wang et al., 2023a) fused dual LiDAR (GEDI/ICESat-2) with optical images, and improved the accuracy of canopy height estimation (R²=0.65~0.90) through the random forest ensemble model, which performed stably in complex terrain and high vegetation coverage areas. Additionally, in response to the demand for global forest canopy height monitoring (Potapov et al., 2021), integrated GEDI LiDAR with Landsat long-term time-series optical data, and used the bagged regression tree algorithm to generate a 30-meter resolution height map. The dataset includes GEDI RH95 indicators, ALS data from multiple locations, and Landsat analysis data. After five-fold cross-validation, the model achieved verification accuracies of R²=0.62 and 0.61 with GEDI and ALS data, respectively, confirming the effectiveness of this fusion method; To focus on tropical forest canopy height estimation (Pourshamsi et al., 2021), fused NASA UAVSAR L-band PolSAR with LVIS LiDAR data, and adopted machine learning algorithms such as RF and RoF. Polarization features were extracted through H/A/Alpha decomposition, and the model was trained with 5000 LiDAR samples. The results showed an average R²=0.70 and RMSE = 10 meters, with sample diversity affecting accuracy, and Subset 1 containing the full height range performing the best, confirming that PolSAR combined with a small amount of LiDAR can efficiently estimate height, providing a new scheme for global forest monitoring; (Yang et al., 2022) used Sentinel-1/2 remote sensing data and 448 quadrat data, and combined random forest algorithm with principal component analysis to achieve plant diversity mapping. The results showed that the predicted R² of Simpson and Shannon-Wiener indices exceeded 0.6, with mapping accuracies of 67.4% and 64.2%, and radar data improved the accuracy of heterogeneity indices by 0.2. This method offers a new approach for large-area plant diversity monitoring and can be extended to global tropical forests in the future.

In the field of biomass and basal area estimation, there are also many achievements (Benson et al., 2021). fused radar, LiDAR, optical data and physical models (MFTM) to achieve high-precision estimation of canopy height (RMSE = 1.68 m) and biomass (RMSE = 1.6 kg/m²) in Canadian boreal forests; (Lahssini et al., 2022) constructed a dual-branch late fusion framework MMFVE based on LiDAR point clouds, Sentinel-2 images and terrain data to estimate the basal area (R² = 0.836) and wood volume (R² = 0.85) of complex forests, verifying the key role of multimodal fusion in improving accuracy. In terms of forest resource deforestation monitoring (Lee and Choi, 2023), aimed to use multimodal satellite images to realize Amazon deforestation estimation, solving the problems of large-area monitoring and weather limitations. The experiment used Sentinel-1, Sentinel-2, Landsat 8 satellite images and monthly mask data to train U-Net series networks (Attention U-Net performed the best), and fused the results with distance similarity. The results showed that this method had high accuracy, providing an effective strategy for Amazon deforestation monitoring.

In general, by integrating LiDAR 3D structure information, optical spectrum/time-series features, terrain data and ground-measured samples, combined with deep learning (such as CNN, self-attention mechanism) or ensemble learning algorithms, it is possible to break through the limitations of a single sensor in penetrability, resolution or terrain adaptability, realize high-precision inversion and long-term dynamic monitoring of forest parameters, supply strong technical support for carbon management, biodiversity conservation and other work, and promote the development of forest ecological monitoring towards a more accurate and efficient direction. Additionally, in tree canopy height inversion, different data sources exhibit distinct advantages and disadvantages(R. He et al., 2024; H. Zhang et al., 2025). Spaceborne data (e.g., GEDI, Landsat) is capable of large-scale and long-time-series observations, enabling canopy height monitoring at the global or regional scale with relatively low costs; however, it has coarse spatial resolution and is susceptible to atmospheric conditions such as cloud cover, which imposes certain limitations on inversion accuracy. Airborne data features higher spatial resolution, allowing for the accurate acquisition of canopy height information with favorable accuracy, yet it incurs higher costs and is restricted by flight platforms and mission planning, resulting in a limited observation range. Unmanned Aerial Vehicle (UAV) data, with the highest spatial resolution, can flexibly obtain small-scale and high-precision canopy height data with excellent accuracy; nevertheless, its cost is affected by factors like flight duration and sensor configuration, and it has a small monitoring scale, making it suitable for local fine-grained research. In practical applications of tree canopy height inversion, it is necessary to reasonably select or fuse different data sources based on factors including research scale, accuracy requirements, and budget. **Table 6** summarizes the research progress of forest structure parameter estimation and ecological monitoring, including information such as data sources, model algorithms, application fields, effects, and publication years of different literatures.

**Table 6:** Research progress in forest structure parameter estimation and ecological monitoring.

Literature number	Data source	Model algorithm	Application area	Effect	Year
(Xiao et al., 2025)	Optical Images, Radar, DEM	ARFCNet	Forest Canopy Height Estimation	The model accuracy has increased by approximately 20% (with R-squared improved by 0.2)	2025
(Ling et al., 2025)	GEDI, ICESat-2, Landsat, Environmental Factors	RF, GBDT, CNN, BP	Forest Canopy Height Estimation	The optimal model has R² values of 0.71 and 0.60 for the training set and test set respectively, with a relative root mean square error of 21.36%	2025
(Benson et al., 2021)	Radar, LiDAR, Optical	MFTM	Forest Canopy Height and Biomass Estimation	Root mean square error of canopy height is 1.68 meters, and root mean square error of biomass is 1.6 kg/m²	2020
(Goel et al., 2025)	GEDI, Landsat, Sentinel-1/2	LSTM	Canopy Height Calculation	The RMSE of the multimodal model is 6% lower than that of the Landsat single modality and 4.7% lower than that of the Sentinel-2 single modality	2025
(Shufan Wang et al., 2023)	GEDI, ICESat-2, Sentinel-2	RF	Forest Canopy Height Estimation	The fused model has an R² of 0.65–0.90 and an RMSE of 0.57–4.15 meters	2023
(Potapov et al., 2021)	GEDI, LiDAR Landsat	Bagged Regression Trees	Forest Canopy Height Estimation	The verification accuracies with GEDI and ALS data are R²=0.62 and 0.61 respectively	2021
(Pourshamsi et al., 2021)	LiDAR, PolInSAR	RF, RoF, CCF, SVM	Forest Canopy Height Estimation	Average R²=0.70, RMSE = 10 meters	2021
(Lahssini et al., 2022)	LiDAR, Sentinel-2, Topographic Information	MMFVE	Forest Parameter Estimation	Compared with the single-modal MLP (L+T), MMFVE increases the total volume R² by 15.3% and the basal area R² by 20.6%	2022
(Lee and Choi, 2023)	Sentinel-1, Sentinel-2, Landsat 8	U-Net Series Models	Forest Deforestation Estimation	The highest F1-score of single-view is 0.841 for Sentinel-1, and it increases to 0.897 after multi-view fusion	2023
(Yang et al., 2022)	Sentinel-1, Sentinel-2, VIs	RF	Forest Diversity Mapping	Predicted R² exceeds 0.6, and mapping accuracies are 67.4% and 64.2%	2022
(R. He et al., 2024)	LiDAR, RGB	ICP,CART	Vegetation parameter extraction	The overall precision rate is increased by 3.7%	2024
(H. Zhang et al., 2025)	LiDAR, RGB	MTCDNet	Individual tree crown detection	AP50 is increased by 94.58%	2025

### 3.4 Forest disaster monitoring and tree health assessment

Frequent forest disasters and potential risks to tree health pose severe threats to the stability of ecosystems and the sustainable utilization of resources (Hoppen et al., 2024). For example, Pine Wilt Disease (PWD) can destroy entire pine forests within 3–5 years, while wildfires directly damage the carbon sequestration capacity and biodiversity of forests. Therefore, accurate monitoring and assessment have become core components of forest protection, and multimodal data fusion technology, with advantages such as information complementarity and strong environmental adaptability, is becoming a key path to break through the bottlenecks of traditional monitoring.

In the field of forest disaster monitoring, the combination of multimodal data and algorithms has significantly improved the recognition efficiency in complex scenarios. To address the challenge of identifying burn scars in the Amazon rainforest (Mohla et al., 2020), used RGB and NIR multimodal satellite images from LANDSAT8 and achieved a training accuracy of 69.51% based on the UNet-based AmazonNET algorithm, providing data support for rainforest ecological damage monitoring for the first time, although there are still challenges in distinguishing interference factors such as rivers. In forest fire early warning, multimodal fusion technology has shown stronger environmental adaptability – the MM-SRENet model proposed in (Jin et al., 2025) fuses smoke images with 12 types of fire risk factors such as temperature and wind speed, achieving a prediction accuracy of 93.06% among 3352 sample pairs including day and night, rain and fog, which is 18.75% higher than that of single-modal models, confirming the key value of multi-source heterogeneous data such as meteorological and topographic data in fire detection(H. Liu et al., 2025; Shaik et al., 2025b).

To meet the full-cycle requirements of wildfire monitoring, multimodal technologies have further expanded application scenarios. (Xiwen Chen et al., 2022) constructed the FLAME2 dataset using UAV RGB/IR bimodal data, achieving a 99.5% wildfire detection accuracy through Early/Late Fusion strategies, among which IR images showed significant robustness in smoke scenarios; (Rui et al., 2023) addressed the challenge of day-night recognition by proposing an RGB-thermal adaptive modal learning network, which improved IoU by 6.41% compared with traditional methods in cross-subset tests, solving the bottleneck of identifying small-scale fire points at night. In addition, (Shaik et al., 2025a) fused L8 optical, SAR and topographic data, achieving a 77% wildfire fuel classification accuracy through the FUELVISION framework, providing a near-real-time multimodal solution for risk assessment; (Bhamra et al., 2023) proposed a multimodal wildfire smoke detection model for scenarios where climate change increases wildfire risks, integrating FIgLib images, weather sensor data and GOES satellite fire point detection, and conducting experiments through the SmokeyNet baseline model, SmokeyNet Ensemble and Multimodal SmokeyNet embedded with weather data, proving that multimodal data can effectively improve detection accuracy and timeliness.

In the field of tree health, multimodal technologies supply precise solutions for pest and disease detection and growth status assessment. Taking Pine Wilt Disease (PWD) as an example, (Lina Wang et al., 2024a) constructed the YOLO-PWD model based on AAV RGB images, improving the AP of discolored pine detection to 95.2% through SE and CBAM attention mechanisms, and the lightweight advantage of the model makes it suitable for large-scale monitoring in epidemic areas; (Feng et al., 2024) took remote sensing images of Pine Wilt Disease in Longyou County, Zhejiang Province, China as experimental data, and proposed the SC-RTDETR framework. Based on RTDETR, it integrates Soft-threshold adaptive filtering and Cascaded-Group-Attention mechanisms, and the model’s mAP is 8.6%-12.9% higher than that of traditional models. This framework has better accuracy and robustness in target recognition in unsafe environments; (Ye et al., 2025) innovatively fused SAR-derived Temporal Moisture Content (TMC) with optical multispectral data, and the developed PWD-Net model achieved an F_1_ score of 0.92 with the support of Sentinel data, breaking through the limitation of optical remote sensing being blocked by clouds. Similarly (Park et al., 2021), used UAV multispectral images (including RGB, NIR and other bands) to construct a multi-channel CNN model, achieving a detection accuracy of 95.48% for dead trees AP, verifying the complementary value of multispectral data in pest and disease identification.

For the monitoring of tree growth status, multimodal technologies also show unique advantages (Pandey et al., 2021). collected visible-near-infrared hyperspectral images of loblolly pine seedlings, and combined Faster R-CNN with SVM algorithms to achieve a 77% accuracy in detecting fusiform rust, which significantly improved the efficiency compared with traditional visual inspection; (Finn et al., 2022) used UAV RGB images to achieve more than 90% accuracy in healthy pine seedling detection through unsupervised machine learning, and no pre-training is required, which greatly reduces the cost of manual annotation; (Lu Wang et al., 2024b) constructed a self-propelled phenotyping platform to collect RGB-D and multispectral images of poplar seedlings, and achieved a 99.69% variety classification accuracy through the ResNet18-CBAM-LSTM model, providing a multi-source time-series data solution for tree health assessment under drought stress.

Based on the relevant case studies of forest fire monitoring and pest/disease monitoring in this section, from the perspectives of monitoring adaptability, cost, and implementability, the “optical data + infrared/thermal infrared data + meteorological data” combination is identified as the core and most cost-effective solution for forest fire monitoring. Specifically, optical data offers ground object texture information, infrared data overcomes interference from illumination and smoke, and meteorological data improves prediction accuracy. These three types of data can be acquired through low-cost UAVs and public databases, covering the entire monitoring cycle of forest fires. For forest pest and disease monitoring (e.g., pine wilt disease), the “hyperspectral/RGB optical data + SAR data + ground measurement data” combination is crucial: hyperspectral data captures physiological stress of trees, SAR data penetrates cloud layers, and ground measurement data enhances model accuracy. Although the cost of this combination is higher than that for fire monitoring, it enables early detection of diseases to avoid greater losses. It is unnecessary to blindly integrate LiDAR into both scenarios, as LiDAR only supplies limited improvement in accuracy while increasing costs significantly. In practical applications, the selection of combinations should be based on specific needs: simplified combinations can be used for regular monitoring to control costs, while full combinations are suitable for key areas to ensure monitoring accuracy. **Table 7** sorts out the research progress in the field of forest disaster monitoring and tree health assessment, covering aspects such as data sources, model algorithms, application fields, effects, and publication years of different literatures.

**Table 7:** Research progress in forest disaster monitoring and tree health assessment.

Literature number	Data source	Model algorithm	Application area	Effect	Year
(Mohla et al., 2020)	RGB, NIR	AmazonNET	Segmentation of Vegetation Burn Scars	Training set accuracy: 69.51%, validation set accuracy: 63.33%	2020
(Jin et al., 2025)	Smoke Images, Temperature, Wind Speed	MM-SRENet	Fire Warning and Risk Assessment	The prediction accuracy reaches 93.06%, which is 18.75% higher than that of the single-modal model	2025
(Xiwen Chen et al., 2022)	RGB, IR Images	Early Fusion, Late Fusion	Wildfire Detection	The accuracy under dual-modality is 99.5%, and the F1 score is 99.56%	2022
(Bhamra et al., 2023)	FIgLib Images, Weather Sensors, GOES Satellites	Multimodal, SmokeyNet	Wildfire Detection	The F1 score is 1.10 higher than that of the baseline model, and the detection time is shortened by 0.64 minutes	2023
(Rui et al., 2023)	RGB, TI Images	Adaptive Modality Learning Network	Wildfire Detection	Compared with the suboptimal RGB-T method, the average IoU is increased by 6.41% and the F1-score is increased by 3.39%	2023
(Shaik et al., 2025a)	L8 Optical Images, S1 SAR, PL SAR, SRTM, FIA	FUELVISION	Wildfire Detection	Overall classification accuracy: 77%	2025
(Pandey et al., 2021)	Hyperspectral Imaging	SVM SMOTE	Fusiform Rust of Loblolly Pine Seedlings	The balanced accuracy of the model based on spectral data of the upper stem reaches 77%, and the AUC is 0.83	2021
(Finn et al., 2022)	RGB Image Data	k-means, Circular Hough Transform	Detection of Healthy Pine Seedlings and Localization of Missing Trees	Detection precision and specificity exceed 90%, and recall rate is nearly 99%	2021
(Lu Wang et al., 2024)	RGB-D, Multispectral Images	LSTM ResNet18-LSM, ResNet18-CBAM-LSTM	Drought Stress Classification	The accuracy of drought stress classification reaches 90.94%	2024
(Feng et al., 2024)	Original Pictures, Pest Attack Images	SC-RTDETR	Pine Wilt Disease	When ϵ=6, the mAP of SC-RTDETR is 12.9% higher than that of RTDETR	2024
(Ye et al., 2025)	TMC, MS	PWD-Net	Pine Wilt Disease	The highest F_1_ score reaches 0.92 when data is fused	2025
(Park et al., 2021)	Multispectral Images, VIs	Multi-Channel CNN	Pine Wilt Disease	The mAP of the multimodal data model reaches 86.63%	2021

## 4 Discussion

Currently, multimodal technology has formed a complete technical system from data collection to intelligent analysis in the field of forest resource monitoring. By integrating multi-source heterogeneous data such as optical, radar, and LiDAR with deep learning algorithms, it realizes complementary fusion of cross-modal information and automatic feature extraction. At the technical method level, multimodal fusion breaks through the limitations of a single data source. Through early fusion, late fusion, and hybrid fusion strategies, it significantly improves the accuracy of information extraction and model robustness in complex forest environments; UAV platforms, relying on high-resolution data collection capabilities, promote the leap of monitoring scales from stand level to individual tree level. Combined with algorithm innovations such as attention mechanisms and cross-modal interaction networks, they realize the collaborative utilization of multi-dimensional features such as spectral texture and three-dimensional structure. In terms of application fields, multimodal technology has made key breakthroughs in forest species classification, carbon storage assessment, pest and disease monitoring, topographic and geomorphic analysis, etc. Through spatiotemporal data integration and dynamic modeling, it supplies systematic technical support for global refined management of forest resources, biodiversity conservation, and ecosystem function assessment, promoting the paradigm transformation of this field from traditional single-modal analysis to multi-dimensional intelligent monitoring.

### 4.1 Main problems and research bottlenecks

#### 4.1.1 Data collection and preprocessing

In forestry multimodal monitoring work, the problems faced in the data collection process are complex and intractable, becoming the primary obstacle to technological advancement. The forest environment is inherently complex, with frequent vegetation occlusion, which often blocks the view of measurement points such as breast diameter, making it impossible to obtain data smoothly; weather conditions such as wind, rain, and fog not only interfere with the quality of images captured by UAVs, making the images blurred, but also reduce the accuracy of LiDAR point clouds, causing deviations in the collected 3D information; unstable GNSS signals in forest areas make positioning and time synchronization extremely difficult, affecting the spatiotemporal consistency of data (Magnuson et al., 2024). The sensors themselves also have many limitations. Passive sensing methods are prone to problems such as image blurring, insufficient image overlap, and poor point cloud penetration due to environmental factors, resulting in collected data failing to accurately reflect the actual situation, which is elaborated in (X. Cheng et al., 2024); LiDAR in active sensing has a range limitation, making it impossible to collect complete data of targets that are too far away, and there will also be repeated tree identification, affecting data accuracy. At the same time, sensor deployment is extremely difficult. Drilling holes in tree trunks to install sensors can easily damage the trees. Environmental interference such as harsh weather and complex terrain in the wild, coupled with sensor range limitations and the high cost required for large-scale deployment, which are mentioned in (Tatsumi et al., 2023), further restrict the breadth of data collection, making it impossible to fully cover large forest areas, and also limiting the depth of collection, making it difficult to obtain more detailed data.

Bottleneck issues in the data acquisition phase can be directly transmitted to the preprocessing stage, thereby significantly increasing the technical difficulty of multimodal data fusion and serving as a core barrier restricting the large-scale implementation of multimodal monitoring technology in forestry. These issues specifically manifest in three key challenges: First, there exists significant spatiotemporal resolution heterogeneity among satellite remote sensing, UAV-borne remote sensing, and ground-based sensors. Systematic biases are easily introduced during geometric registration and temporal synchronization, resulting in the disruption of feature correlation across multimodal data and hindering the effective fusion and complementation of multi-source information. Second, complex environments severely limit the stability of data quality. Optical remote sensing and LiDAR data are vulnerable to cloud cover, rain-fog interference, and vegetation canopy obstruction, leading to data missing or structural biases. Although the inherent speckle noise in SAR images can be mitigated through filtering preprocessing, it cannot be completely eliminated, which continuously interferes with subsequent feature extraction and model training. Third, the bottlenecks of small-sample size and data annotation are particularly prominent. The scarcity of samples for specific targets (e.g., rare tree species) tends to cause model overfitting and insufficient generalization ability, making it difficult to adapt to actual monitoring scenarios. Additionally, manual annotation is associated with high costs and low efficiency, while the pseudo-labels generated by weak-supervision strategies in semi-supervised learning also have inherent errors, further reducing the stability of model training. These bottlenecks restrict the application of multimodal monitoring technology in forestry at the data foundation level, and there is an urgent need to break through the full workflow of “data acquisition-preprocessing-analysis and application” via technological innovation.

#### 4.1.2 Multimodal data fusion strategies

In the process of applying forestry multimodal data, multimodal data fusion strategies also face many difficulties, which restrict their accurate application in various scenarios. Different modal data (optical, radar, LiDAR, etc.) have significant differences in feature representation, distribution, and statistical characteristics, with heterogeneity, making it difficult to directly conduct fusion analysis. This heterogeneity makes it difficult for the model to effectively explore the correlation and complementarity between modalities, which not only reduces the fusion effect but also may cause overfitting or underfitting problems, negatively affecting the generalization ability of the model. The selection of fusion methods is also extremely critical. Early fusion requires the design of complex feature fusion algorithms, which easily causes feature redundancy and increases the learning burden of the model; although late fusion avoids feature redundancy to a certain extent, it complicates the decision-making process, making it difficult to fully utilize modal complementarity. Moreover, for special forestry scenarios (such as complex forest environments, diverse tree species, and pest characteristics), there is a lack of highly universal fusion strategies that cannot flexibly adapt to different forestry monitoring needs. At the same time, there are inconsistencies and incompleteness at the data level. Different data sources have differences in the description of forest parameters and tree species characteristics. Some modal data may be incompletely collected due to environmental factors (cloud occlusion, vegetation coverage), which further increases the difficulty of fusion. However, existing technologies have difficulty balancing accuracy, efficiency, and cost when dealing with these problems, which greatly restricts the effectiveness of multimodal data fusion in precise forestry applications.

Most existing deep learning fusion algorithms are developed for a single field (such as infrared-visible), lacking cross-domain universality and unable to adapt to the needs of multiple forestry scenarios. Moreover, pure CNN or Transformer structures have inherent defects, making it difficult to balance local and global information, which affects the extraction and fusion of complex forestry features (Zhao et al., 2024). In terms of cross-modal feature interaction, early fusion (such as directly concatenating spectral and LiDAR features) ignores the nonlinear relationships between modalities, which is prone to cause the “curse of dimensionality”. When the number of features exceeds 500 dimensions, the classification accuracy will decrease by 12%; late fusion (such as random forest weighting) lacks deep semantic correlation, and its improvement effect on complex forestry scenarios (such as soil erosion identification in undulating terrain areas) is limited, only 8%-10% higher than that of single-modal methods. Multimodal fusion also faces a series of technical challenges. In feature alignment, the feature space differences between different modalities (such as RGB and thermal infrared) are large, and cross-modal mapping needs to be established through methods such as CCA; in terms of noise robustness, if sensor failure causes data distortion in a certain modality, it is necessary to deal with uncertain information with the help of Dempster-Shafer theory; in terms of computational complexity, when integrating video-text-sensor data, the computational load of traditional fusion methods increases exponentially with the number of modalities, which needs to be optimized by lightweight networks (such as MobileNetV2), but this may sacrifice part of the accuracy(K.-L. Du et al., 2025). In addition, the model has weak generalization ability. When existing algorithms (such as UNet) are applied across regions (from the Amazon rainforest to subtropical forests in China), the accuracy will drop significantly (OA from 91.49% to 73.2%) due to factors such as spectral differences. Transfer learning also requires additional annotation of 20% of the target area samples, which increases the application cost and difficulty. In addition to transfer learning, domain adaptation technology can address the generalization issue more precisely: For the “spectral domain shift” in forestry (e.g., differences in vegetation spectral characteristics between temperate and subtropical forest areas), feature-level adversarial training can extract domain-invariant features, improve cross-regional classification accuracy, and does not require target domain annotations. For the “modal quality domain shift” (e.g., differences in LiDAR point cloud density across different forest areas), modal-level domain adaptation can supplement low-quality modal features to mitigate accuracy fluctuations. In the future, optimizing the loss function by incorporating forestry ecological priors will further enhance its adaptability to forestry scenarios. These problems are intertwined, and from data heterogeneity, method adaptation, technical challenges to generalization ability, they comprehensively hinder the in-depth application and efficiency improvement of multimodal data fusion strategies in forestry monitoring, and targeted technical breakthroughs are urgently needed.

#### 4.1.3 Model deployment and application level

In the model deployment and application stage of forestry multimodal monitoring, a series of complex and critical issues need to be addressed urgently, which severely restrict the transformation of technical efficiency into actual production. From the perspective of model lightweight adaptation, the contradiction between the scale of deep learning models and the resource limitations of edge computing devices(S. J. Ma et al., 2024; Nie et al., 2025) is prominent. Currently advanced multimodal fusion models, in pursuit of high accuracy, often have a huge number of parameters. However, UAVs and ground edge terminals commonly used in forestry monitoring have limited memory and computing power resources due to hardware costs and original design intentions. This makes it difficult to directly deploy the models. Even if they are barely adapted, problems such as running lag and significant delays will occur due to hardware performance bottlenecks, which cannot meet the needs of “low latency and high response” in scenarios such as forest fire early warning and real-time monitoring of pests and diseases, greatly reducing the practical value of the technology in actual forestry management. In terms of model generalization and multi-task adaptation, the complexity of forestry scenarios is far greater than that of conventional environments. There are significant regional differences in forest ecosystems, ranging from tropical rainforests to cold temperate coniferous forests, and from plain forest areas to mountain forest areas, with vastly different vegetation types, topographical conditions, and climatic characteristics. After a single model is trained, its generalization ability drops sharply when applied across regions and scenarios in the face of new spectral features and topographical structures. At the same time, forestry monitoring tasks are diverse, from canopy height inversion and biomass estimation to pest identification and fire risk assessment. Different tasks have different requirements for data features and model outputs. Existing models are difficult to meet the needs of multiple tasks, and the cost of retraining when switching tasks is high and the cycle is long, resulting in severe insufficient system flexibility and inability to efficiently support the dynamic and diverse monitoring needs in forestry management.

### 4.2 Countermeasures

In response to the three core problems in the above practice: first, the high cost of data acquisition and high difficulty of preprocessing, as sample annotation relies on professional human resources and the differences in multimodal data formats lead to low integration efficiency; second, the insufficient adaptability of deep fusion architectures, where existing algorithms struggle to balance the complementarity and redundancy of multimodal features, limiting the generalization ability of models; third, the difficulty in the implementation of scenario-based applications, as technology is disconnected from actual business needs and there is a lack of systematic integration solutions. This section will propose targeted countermeasures from three dimensions: “Optimization of Data Acquisition and Preprocessing”, “Innovation of Deep Fusion Architectures and Algorithms”, and “Scenario-Based Application and System Integration”, so as to supply solutions for the efficient implementation of multimodal data fusion technology.

#### 4.2.1 Optimization of data acquisition and preprocessing

To address the core bottlenecks in the process of forestry multimodal monitoring—including technical obstacles in the full workflow of data acquisition and preprocessing, small sample sizes, and high costs caused by data annotation—a coordinated solution system integrating “basic workflow optimization + algorithmic strategy breakthrough” must be established. In the data acquisition phase, to tackle the complex forest environment, limitations of sensor performance, and high deployment costs, an environment adaptation scheme combining “multi-temporal observation + multi-path GNSS enhancement + UAV collaborative flight” is adopted, along with real-time fusion technology for LiDAR and optical imagery. Meanwhile, implementation costs are reduced by developing occlusion-resistant and high-penetration sensors, integrating active-passive sensing, and innovating non-intrusive distributed deployment strategies (Ehrlich-Sommer et al., 2024). In the dataset construction phase, aiming to solve the problems of small sample sizes (e.g., for rare tree species), high annotation costs, and data silos, scarce samples are expanded via cross-modal generation and transfer learning techniques, and an industry-wide data sharing platform is built to break down data barriers. The combined “weak supervision + active learning” model is used to lower annotation costs, pre-trained models are introduced to accelerate the processing workflow, and a data validation model is constructed to ensure data quality. A typical example is the 339 km² PureForest multimodal dataset built in (Gaydon and Roche, 2024), which includes airborne LiDAR point clouds and ultra-high-resolution imagery covering 13 semantic classes; this dataset has verified the complementarity between the LiDAR modality (OA = 80.3%) and the imagery modality (OA = 73.1%). In the preprocessing phase, to resolve difficulties in spatiotemporal alignment and environmental interference on data quality, the “tree-oriented geometric alignment + temporal LSTM imputation” technique is employed to address spatiotemporal heterogeneity and data missing. The original quality of each modality is improved through customized workflows, and lightweight modules are embedded to achieve pre-fusion of complementary features. For the core algorithmic challenges of small sample sizes and high annotation costs, two optimization strategies are adopted: active learning reduces annotation workload through the “uncertainty sampling - diversity selection” approach, while semi-supervised learning compresses annotation costs to 1/4 of those of fully supervised learning by leveraging “a small amount of annotated data + a large amount of unannotated data” combined with pseudo-label generation and optimization techniques. In the future, it will be necessary to further integrate the spectral-structural coupling features of trees, optimize sample selection mechanisms and pseudo-label error control strategies, and enhance the adaptability of algorithms to forestry scenarios.

#### 4.2.2 Deep fusion architecture and algorithm innovation

To address the three core issues identified in multimodal data fusion strategies: “rigid weighting allocation of unimodal features leading to insufficient utilization of complementarity, separation of local features and global information impairing the integrity of fused representations, and low alignment accuracy of multimodal features in complex forest stands”, targeted solutions can be proposed from the perspective of deep fusion architectures and algorithms. To solve the problem of rigid weighting, we can draw on the concept of the Dynamic Modality Scaling (DMSM) module designed in (Vahrenhold et al., 2025) for multi-source single-tree LiDAR point cloud classification; this module adaptively adjusts weights according to the feature quality of different modalities such as LiDAR point clouds and Full-Waveform (FWF) data, achieving an Overall Accuracy (OA) of nearly 97% in multi-source single-tree LiDAR point cloud classification tasks, and based on this, a dynamic modal weight adaptive mechanism can be designed, which quantifies feature effectiveness by calculating indicators like LiDAR point cloud density and spectral clarity of optical images, automatically allocates weights to each modality, and avoids the limitation of feature complementarity caused by fixed weights. To integrate local and global information (Zhao et al., 2024), innovatively adopts a hybrid Transformer-CNN encoder structure, using CNN to extract local detail features of images and Transformer to capture global information correlations, and combined with a composite attention fusion strategy (including axial attention and channel attention modules), its efficient information integration capability has been verified on 6 datasets covering infrared-visible, multi-exposure, and medical images; based on this, the study can construct a hybrid encoding architecture of “CNN extracting local features of single-tree texture/canopy details + Transformer modeling global correlations of stand spatial distribution”, and integrate the composite attention module to enhance the interaction between local and global information. To tackle the issue of feature alignment in complex forest stands, by leveraging the advantages of the aforementioned architectures, precise feature alignment can be achieved through two-dimensional optimization: “temporal regularization (controlling the acquisition time difference between optical and LiDAR data to ≤ 24 hours) + semantic binding (taking individual trees as anchors to associate LiDAR tree height parameters with optical vegetation indices)”, thus forming a deep fusion solution suitable for forestry scenarios.

#### 4.2.3 Scenario-based application and system integration

To address the challenges in the deployment and application of forestry multimodal models, scenario-based applications and system integration offer solutions from multiple dimensions. For example, building an end-to-end multi-task network to simultaneously output indicators such as canopy height and pest severity, reducing redundant calculations and adapting to multi-task requirements; creating an integrated “space-air-ground” monitoring platform that combines satellite-based macro trend analysis with UAV-based local fine detection to meet the needs of different accuracy and scale in scenarios such as forest fire early warning, alleviating the problem of model generalization across regions/scenarios; integrating physical models (such as the fractal tree model MFTM (Benson et al., 2021)) with data-driven models, using physical prior constraints (such as limiting the prediction range of canopy height) to improve the reliability of parameter inversion in complex forests (mountain cloud forests) and enhance the applicability of models in heterogeneous environments. Two types of core verified physical models in the forestry field can be prioritized for integration: First, ecological process models, such as the CENTURY model that characterizes forest carbon cycles and nutrient balance, and the BIOME-BGC model that simulates vegetation-climate-soil interactions. By embedding the ecological mechanisms contained in these models (e.g., the quantitative correlation between photosynthetic rate and spectral reflectance) into the multimodal data-driven framework, the errors in carbon stock estimation caused by cloud cover and sparse vegetation can be reduced. Second, tree growth models, such as the 3-PG model based on physiological processes and the LIGNUM model focusing on individual tree growth dynamics. Through their coupling formulas for tree height, diameter at breast height (DBH), and environmental factors (temperature, precipitation), the weight allocation of LiDAR structural parameters and optical spectral features can be optimized, thereby improving the accuracy stability of biomass inversion throughout the entire growth cycle from young forests to mature forests. researching multimodal sensors to promote the transformation of forestry towards intelligence through a “perception-decision-execution” closed loop (Pereira et al., 2023). Meanwhile, from the perspective of model lightweighting, indirectly alleviating the resource constraints of edge devices through methods such as multi-task unified modeling, helping models to be efficiently deployed and applied in forestry scenarios, and promoting the transformation of technology into actual production.

## 5 Conclusion and outlook

Multimodal data fusion technology has established a comprehensive technical system in the field of forest resource monitoring. Its core lies in integrating multi-source data (e.g., optical, radar, and LiDAR data) with deep learning algorithms to achieve cross-modal information complementarity and automated feature extraction. The specific technical workflow exhibits a hierarchical optimization characteristic: at the data acquisition layer, by relying on a “space-air-ground” collaborative network, it integrates diverse data sources such as satellite remote sensing, UAV-borne payloads, and ground-based sensors, effectively overcoming the spatiotemporal coverage limitations of a single data source; at the preprocessing stage, key operations including data cleaning, spatiotemporal registration, and feature dimensionality reduction lay a high-quality data foundation for subsequent fusion analysis; at the fusion technology layer, it not only adopts feature-level and decision-level fusion strategies of traditional machine learning but also employs early/late/hybrid fusion schemes of deep learning, which effectively addresses the issue of modal heterogeneity. Particularly, supported by UAV platforms, it has realized a leap in monitoring accuracy from the stand scale to the individual tree scale. This technology has covered core scenarios in the forestry field and achieved remarkable results: in tree species classification, it improves accuracy through the fusion of spectral and 3D structural features; in land resource monitoring, it optimizes soil moisture prediction and land cover classification performance by combining multi-source data; in forest structure parameter estimation, it retrieves canopy height and biomass using LiDAR and optical data; in disaster and health assessment, it integrates multimodal information to realize wildfire early warning and pest/disease detection. In the sub-scenario of urban forestry, it further combines UAV RGB/LiDAR data with street-view images to complete individual tree segmentation and tree species classification of street trees, and incorporates environmental data (e.g., urban heat island, traffic noise). Through deep learning models, it retrieves the ecological benefits of cooling and humidification provided by urban forests. Overall, relying on core advantages such as information complementarity, model generalization ability, and ecological management empowerment, this technology is driving the transformation of forestry monitoring from traditional models toward intelligent and precise directions.

Current research encounters bottlenecks in multiple aspects: Data acquisition is constrained by the complexity of forest environments (e.g., vegetation occlusion, weather interference) and sensor performance (e.g., LiDAR range, passive sensing quality); registration errors arise during preprocessing due to differences in spatiotemporal resolution; and model training is limited by small sample sizes and high annotation costs. In terms of fusion strategies, there are significant disparities in modal feature spaces, existing algorithms lack cross-domain universality, and accuracy declines due to spectral differences in cross-regional applications. For model deployment, there exists a conflict between the large parameter volume of deep learning models and the limited resources of edge devices; adaptation to multiple tasks requires retraining, resulting in insufficient flexibility and real-time performance. Targeted breakthroughs in future research can be achieved in the following aspects:

Direction for Optimization of Data Acquisition and Preprocessing. To address issues such as reduced LiDAR point cloud accuracy caused by complex forest environments, tree damage from sensor deployment, and spatiotemporal registration errors during preprocessing, two measures are proposed. On one hand, develop an “anti-interference multimodal sensor integration technology” that combines miniaturized LiDAR with high-penetration hyperspectral sensors. When matched with the low-altitude hovering and obstacle avoidance mode of unmanned aerial vehicles (UAVs), this technology minimizes the impact of vegetation occlusion. Meanwhile, develop non-invasive bark-attached sensors to avoid trunk damage. On the other hand, construct a “Transformer-based spatiotemporal registration model” that automatically matches the spatiotemporal features of satellites (e.g., Sentinel-2), UAVs (sub-meter resolution), and ground sensors. This model reduces registration errors caused by resolution differences, laying a high-precision data foundation for subsequent fusion analysis.

Direction for Innovation of Multimodal Fusion Strategies. Aiming at problems including modal heterogeneity (mismatched feature spaces between optical data and LiDAR), superficial fusion strategies, and weak cross-regional generalization, three solutions are put forward. First, propose a “meta-learning driven cross-modal feature mapping algorithm” that dynamically adjusts the feature spaces of optical data, LiDAR, and SAR to resolve heterogeneity issues. Second, develop a “dynamic dimensionality reduction - cross-attention fusion framework”: embed a dynamic dimensionality reduction module for high-dimensional features in the bottom convolutional layer (to avoid the curse of dimensionality) and strengthen the semantic association between modalities through cross-attention in the upper fully connected layer. Third, construct a “domain adaptation model embedded with ecological priors” that integrates knowledge of forest types and climate zones. Through self-supervised learning, this model adapts to cross-regional spectral differences, ensuring that the reduction in classification accuracy is less than 5%.

Direction for Model Deployment and Application Implementation. To tackle problems such as a 20%-30% increase in inference latency of edge devices and the need for retraining when adapting to multiple tasks, two approaches are adopted. First, develop a “lightweight model dedicated to forestry”: compress the parameters of models such as MMTSCNet and TSCMDL by 40%-60% based on knowledge distillation, and deploy the compressed models with FPGA chips to reduce the inference latency of UAV terminals to less than 1 second. Second, design a “unified multi-task modeling framework” with Transformer as the backbone. Through a “shared feature layer + task-specific output head” structure, this framework enables simultaneous inference for “tree species classification - canopy height inversion - forest fire early warning” without retraining, improving the flexibility of the system. In addition, the digital twin can also be combined with multimodal fusion technology to try to construct a digital twin of forestry scenarios and integrate multimodal fusion technology into it, so as to achieve more accurate simulation, prediction and management of forest ecosystems, and offer new ideas and methods for the intelligent development of forestry.
